# Topological Frustration Triggers Ultrafast Dynamics of Monolayer Water Confined in Graphene Slit Pores

**DOI:** 10.1021/acs.nanolett.4c04077

**Published:** 2024-11-26

**Authors:** Banshi Das, Sergi Ruiz-Barragan, Biman Bagchi, Dominik Marx

**Affiliations:** † Lehrstuhl für Theoretische Chemie, Ruhr-Universität Bochum, 44780 Bochum, Germany; ‡ Departament de Fisica, Universitat Politecnica de Catalunya, Rambla Sant Nebridi 22, 08222 Terrassa, Barcelona, Spain; ¶ Solid State and Structural Chemistry Unit, Indian Institute of Science, Bangalore 560012, Karnataka India

**Keywords:** Hydrogen Bond, Dangling Bond, Ultrafast Diffusion, Nanoconfined Water, Topological Frustration

## Abstract

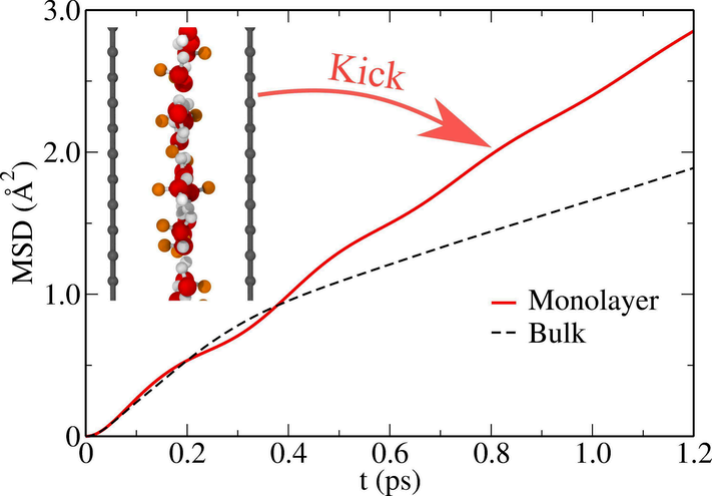

Nanoconfined water exhibits astonishing properties that offer new opportunities in physics, biology and technology like energy-storage applications. Here we study such nanoconfined water using *ab initio* molecular dynamics simulations to elucidate the structure and dynamics of water monolayers in graphene-based slit pores. The significant population of dangling (or free) O–H bonds pointing toward the two confining walls, leads to topological frustration in the hydrogen bond network. This provides a novel channel for ultrafast diffusion distinct from what has been observed in bulk or interfacial water.

Altered dynamics of water in nanoconfined environments is of fundamental importance for physics, chemistry and biology. Water in such confined situations offers opportunities in many technologies including energy storage^1,2^ or water desalination^3,4^ to name but a few. Amazing effects have been observed long ago for one-dimensional transport of water in aqueous solution involving carbon nanotubes.^5−9^ Much more recently, controlled planar nanoconfinement has been achieved experimentally with nanofluidic slit pore setups.^10−16^ This opened new opportunities to examine two-dimensional water in the bilayer and even in the monolayer regime, with control of the pore width at the Å-scale due to these significant experimental advances. Most interestingly, in the limit of extreme confinement when water is squeezed into a monolayer lamella between two graphene walls kept at an interlayer distance below 10 Å, water is found to behave strikingly differently as compared to both bulk and interfacial water.^10,12,13,15,17−25^ Graphene-based slit pores are broadly used in the experimental as well as computational literature to investigate confinement effects since chemically complex materials such as hydrophilic walls would additionally lead to strong H-bonding interactions absent with graphene. In graphene nanoconfinement, the altered, often peculiar properties of water may be understood fundamentally in terms of the strongly perturbed H-bond network of water itself that is enforced within such severely restricting geometries. However, any mechanistic understanding of the ultrafast dynamics of monolayer water is still lacking.

Previous experiments as well as molecular dynamics simulations have already revealed a surprising ultrafast flow of water monolayer sandwiched between two graphene walls.^10,13,15,19,23^ Yet, despite the tremendous implications in fundamental and applied science, as mentioned above, the molecular mechanism in terms of H-bond rearrangements within monolayer water that causes such ultrafast dynamics still remains unknown. Relaxation dynamics of water is well-known to take place through rearrangements of the H-bond network, both in bulk^26−35^ and at interfaces.^33,36−39^ The extended jump mechanism,^28^ where a reorienting water molecule changes its H-bonding partner by a large-amplitude angular jump, has been found to be remarkably successful for bulk^29,31,33^ and interfacial water.^33,36,39^ Notably, it has been shown that this very mechanism also explains diffusion in bulk water.^34^ It is interesting to note that despite the increased heterogeneity at aqueous interfaces, such as the presence of a small fraction of free O–H (or dangling) bonds at hydrophobic interfaces, the reorientation dynamics at these interfaces has been shown to be still governed by H-bond exchanges, involving now tangential or straddling O–H bonds^33,36,39^ much like observed in bulk water.

Recent theoretical studies reveal molecular details of the unique structural aspects of water at extreme confinement, for instance in H-bond populations and electronic properties,^17,40,41^ pronounced anisotropic dielectric response,^18,42,43^ characteristic IR,^41^ THz,^21^ Raman,^44^ and vibrational sum frequency generation^24,25^ spectra. These have been found to be strongly different from both, bulk and interfacial water. While similar mechanistic frameworks are now firmly established to describe the structural dynamics of bulk and interfacial water,^28,29,31,33,34,36,39^ such a framework is yet lacking to explain the ultrafast dynamics of monolayer water at extreme confinement.

In the present study we use *ab initio* based MD simulations^45^ (see Supplemental Sec. 1 for details), to unravel the mechanism of ultrafast water diffusion in the confined monolayer limit. We find that the underlying mechanism is completely different from that of the bulk. When subjected to extreme confinement in graphene slit pores, water molecules do not have the opportunity to form the four H-bonds common in bulk water. Instead, a significant population of O–H bonds remains free and thus dangling. This leads to pronounced topological frustration unseen in both, thicker confined water lamellae and at wide interfaces. As worked out below, it is these frustrated dangling O–H bonds generated upon monolayer confinement that open a new channel for “kick-enhanced diffusion” – unknown from bulk and interfacial water–which produces substantially enhanced mobility within *persisting* solvation shells, i.e. even without any exchange of H-bonded partners in the solvation shells as known from bulk and interfacial water. In the present work, we mainly discuss the mechanism of translational diffusion, which is associated with the rearrangement of the H-bond network and is also closely coupled to rotational dynamics. Thus, the mechanistic framework presented here is broadly applicable to explain the ultrafast dynamics as such of monolayer water.

The three-dimensional (3D) extended H-bond network is widely acknowledged to be responsible for the unique properties of bulk liquid water. At extreme confinement in narrow slit pores, when only a single-layer water lamella can be hosted due to the geometric restriction, there is no opportunity for water to establish such 3D H-bond networks. This leads to topological frustration as discussed below. As depicted in Figure 1(a), *all* water molecules in such a monolayer form H-bonds with three or only two neighboring molecules, in stark contrast to mostly four in the bulk (see Supplemental Figure S2 for the definition of H-bond). This extreme confinement effect leads to two different kinds of O–H states: (i) One is H-bonded (HB) with another water molecule and mostly oriented in the plane of the graphene walls, and (ii) the other remains free, yielding a dangling bond (DB). Importantly, these DBs originate from the same single water layer and point either toward one or the other confining wall (see the snapshot in Figure 1(a) where the DBs stick out of the lamella toward the left and right graphene sheets).

**Figure 1:**
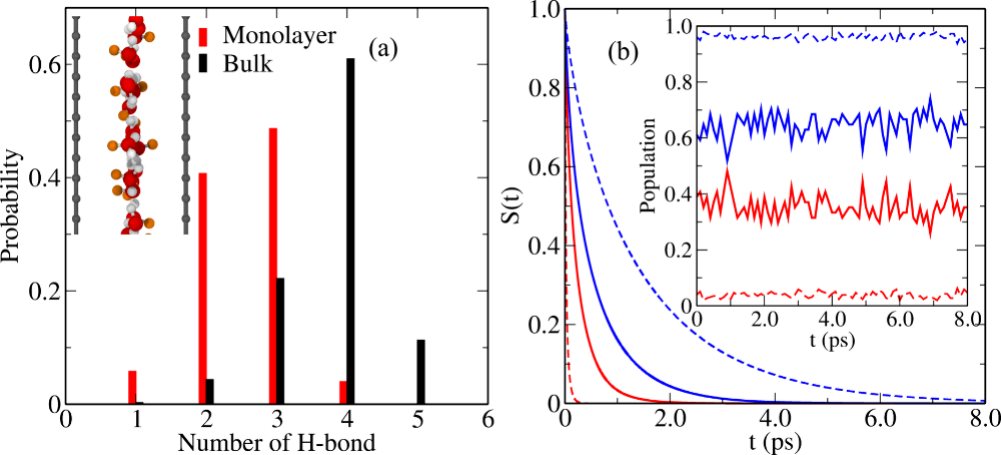
(a) Hydrogen bond populations in confined monolayer (red) and bulk (black) water; a representative snapshot of monolayer water confined between two graphene walls is shown in the inset. (b) Continuous time correlation functions of dangling bond (DB, red) and hydrogen bond (HB, blue) states for confined monolayer (solid) and bulk (dashed) water; the representative time evolution of the DB/HB populations is shown in the inset for a typical trajectory segment.

The average lifetime of HB (or DB) states (τ) is extracted from the integration of continuous time correlation function, *S*(*t*)=⟨*h*(0)*H*(*t*)⟩/⟨*h*⟩, where *h*(*t*) is 1 if O–H is H-bonded (or dangling) at time *t*, otherwise 0, and *H*(*t*) is 1 if O–H remains continuously H-bonded (or dangling) from time 0 to *t*, otherwise 0 (see Figure 1(b)). In the monolayer, the DB states are found to relax approximately two times faster (τ = 0.24 ps) compared to the relaxation of HB states (τ = 0.52 ps). This picture is very different in the bulk, where DB states relax ∼46 times faster (τ = 0.03 ps) than the HB states (τ = 1.38 ps).

The monolayer water contains a significant fraction of DB states leading to severe topological frustration in the residual H-bond network, while in bulk, the occurrence of DB states is rare since those are formed only transiently as short-lived fluctuations within stable H-bonded states or during the fleeting exchange of H-bond partners. This is demonstrated in the inset of Figure 1(b), where a significant fraction of O–H in monolayer water remains in the DB state (≈0.35) in contrast to the negligible fraction (≈0.04) found in bulk. As the DB states appears transiently in the bulk, they need not be included as separate states, unlike the HB states. On the other hand, the geometric constraints on H-bonding in the monolayer prevent the O–H group to donate stable H-bonds, resulting in a significant population of O–H bonds that remains in the DB state, which in turn makes the HB state more labile than in the bulk liquid. This observation will be crucial for explaining the mechanism of ultrafast diffusion as illustrated below.

In the bulk, a water molecule can reorient and diffuse by a substantial amount when one of its existing donated H-bonds breaks. This can happen when a new H-bond with another acceptor oxygen atom forms and an old bond breaks. At monolayer confinement, the H-bond rearrangement process is more involved due to the additional lability of H-bonds. The later, as already mentioned, originates from the significant population of frustrated dangling O–H bonds, shown in Figure 1(b). In particular, when a specific donor–acceptor H-bonded pair breaks its H-bond, the *same* water pair gets rebonded with a *different* donor–acceptor combination (see Supplemental Figure S3). Such a process involves both, net displacement and rotation. This gives rise to a fast new channel of H-bond breaking and reformation dynamics *without* exchanging the H-bonded partner molecule, which is dominant in the case of bulk water.^28,29,34^

Inspired by earlier works,^29,46^ we now propose a novel stable state based description that provides a general platform to capture the mechanistic features of diffusion in H-bonded environments. It naturally encompasses the standard situation with long-lived H-bonds (like in bulk or interfacial water) as a special case. Additionally, it enables us to describe the scenario of floppy H-bonded water in confined monolayers. We first assign the stable state of water as its most populated coordination state, which is the three-coordinated solvation shell for the monolayer and the four-coordinated one in bulk water. After filtering out any transient fluctuation within a stable state on ultrashort time scales, we can identify all transition events between those states, see Supplemental Sec. 4 for the details.

With this solvation shell-based description of stable states (which is evidently different from the earlier one based on H-bonded pairs^29,34,36^), we first trace all exchange events from one such stable state basin (meaning a particular solvation shell) to another one (and thus another solvation shell composition) and refer to this process as “solvation water exchange” (SWE). In this process, a water molecule (W*) moves from one basin to another one when one of its solvation shell members (which we denote by Wa) is replaced by another water molecule (called Wb). The time origin of the SWE event, marked as *t* = 0 in Figure 2(a) where we provide key analysis of this process, is defined when the oxygen atoms of Wa (denoted by Oa) and Wb (i.e., Ob) are equidistant from the oxygen of W* (i.e., O*), see the representative snapshot sequence (c), (d) and (e) of Figure 2. As depicted in Figure 2(a), in the confined monolayer system, the O*–Oa (or O*–Ob) separation distance is significantly greater after (or before) the transition events compared to what happens in bulk. The average O*–Oa (O*–Ob) distance within the stable state basin, i.e, before (or after) the SWE transition, is longer in the monolayer. This suggests high flexibility of H-bonds within solvation shells in monolayer water, leading to large displacements (δ_O*_) of O* in the solvation shell in the confined monolayer as depicted in Figure 2(f). This remarkable flexibility of the solvation shell and large distance separations after SWE events are also evident in the O–O radial distribution function and potential of mean force as depicted in the supplemental Figure S1.

**Figure 2:**
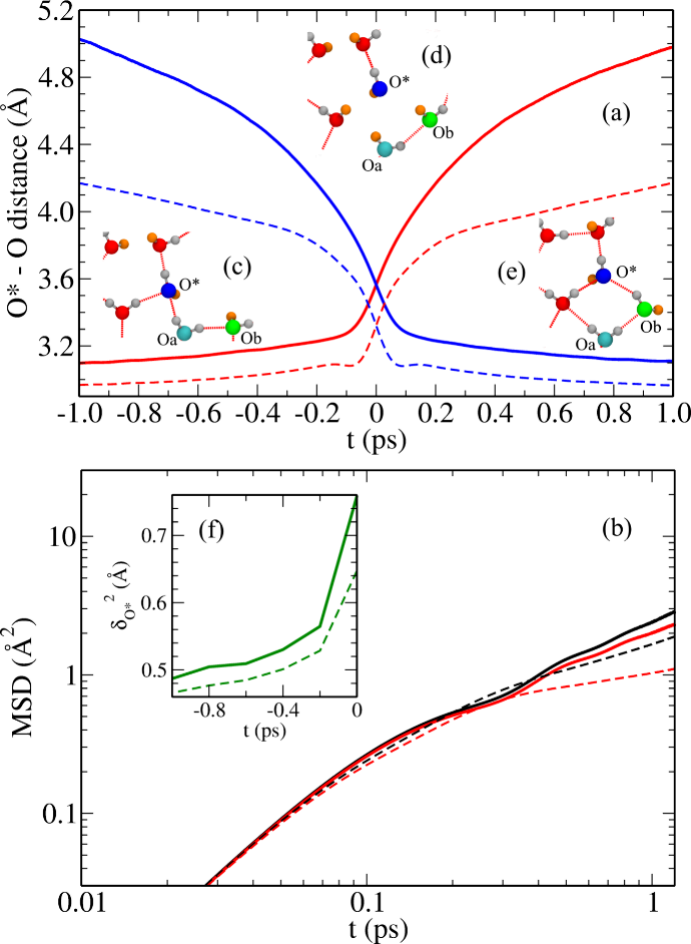
(a) Change in the O*–Oa (red) and O*–Ob (blue) distances, see the text and figure insets, during solvation water exchange (SWE) events for confined monolayer (solid) and bulk (dashed) water. Insets (c), (d) and (e) are representative snapshots before, during and after the exchange, respectively, where the O*, Oa and Ob oxygens are shown in blue, cyan and green whereas the DB hydrogens are shown in orange instead of gray. (b) Mean-square displacement (averaged over all molecules) in confined monolayer (black solid) and bulk (black dashed) water and MSD of water molecules in a solvation shell in confined monolayer (red solid) and bulk (red dashed) water. (f) Mean-square displacement amplitudes of O* (δ_O*_^2^), see text, in the course of SWE events in confined monolayer (green solid) and bulk (green dashed) water.

Importantly, the contribution of SWE processes toward the total translational diffusion of a water molecule (W*) can be quantified in terms of the continuous time random walk (CTRW) model,^34,47^ as *D*_SWE_=δ_O*_^2^/2*dτ*_SWE_, where τ_SWE_ is the SWE time, δ_O*_ is the displacement amplitude and *d* is the dimension of the system (see Supplemental Sec. 6 and 7 for background and definitions). The SWE time is calculated from the time integration of the jump-time correlation function^29,34^ as [1-⟨*p*_i_(0)*p*_f_(*t*)⟩], where *p*_i_(*t*) (or *p*_f_(*t*)) is 1 before (or after) the exchange event and 0 otherwise. The residual diffusion *D*_frame_ (commonly called “frame contribution”) is calculated from the mean-square displacement (MSD) of O* (see Supplemental Sec. 8 for details) within a specific solvation shell, see Figure 2(b). Thus, *D*_frame_ quantifies the diffusion of solvated water complexes together with O*, whereas *D*_SWE_ is the additional contribution due to solvation water exchange processes involving O*. For bulk diffusion, we find SWE events to contribute predominantly (here *D*_SWE_ ≈ 1.42 × 10^–5^ cm^2^s^–1^) to the total diffusion coefficient compared to frame diffusion (here *D*_frame_ ≈ 0.63 × 10^–5^ cm^2^s^–1^), which is fully consistent with earlier observations for bulk water,^34^ see Supplemental Sec. 8 for details.

However, in stark contrast with the above scenario in bulk water, the dominant term in self-diffusion within confined monolayer water is found to be frame diffusion (*D*_frame_ ≈ 4.06 × 10^–5^ cm^2^s^–1^), with only a minor contribution from the solvation water exchange dynamics (*D*_SWE_ ≈ 1.45 × 10^–5^ cm^2^s^–1^). Similar to that of bulk, we also find that the diffusion of water molecules at the usual graphene water interface mostly originates from the SWE events (see Supplemental Sec. 11). This highlights the unique role of H-bond fluctuations within the *same* solvation shell basin, even before any of the solvation shell members are exchanged, which is completely different from bulk water as further demonstrated below.

In monolayer confinement, the frustrated dangling O–H bonds enable ultrafast translational dynamics of a water molecule even before any SWE event occurs as just discussed. A similar channel is almost totally absent for diffusion in bulk water, where a water molecule has to “wait” for an SWE event to occur in order to translate in space.^34^

To further understand and quantify the origin of the enormous mobility of water within a nanoconfined solvation shell, we analyze the transitions of O–H bonds from HB to DB states within the same solvation shell basin. Each such transition displaces the initially H-bonded pair far apart, like a “kick”, yet the solvation shell remains intact as the water pair subsequently reforms a H-bond via the same or a different donor–acceptor combination (see quantitative analyses in Supplemental Figure S3). This is due to the broadened first solvation shell in the confined monolayer as quantified by the O–O radial distribution function, requiring a higher free energy to escape from the shell compared to bulk (see Supplemental Figure S1). Thus, in a particular solvation shell, a water molecule (O*) experiences kicks from all the three HB pairs (given the predominantly 3-fold H-bonding in the monolayer). The time origin for that specific HB-to-DB transition event is considered to be the time when that tagged H-bond breaks (see Supplemental Sec. 5). The O*–O distance averaged over all such events is depicted in Figure 3(a) along the respective HB-to-DB transitions, where what we call “kick” event happens around *t* = 0 ps. The translational displacement is found to be associated with a specific change of orientation of that O–H bond, which has to be oriented in the plane of the confining walls in HB states but becomes perpendicular to the walls when reaching the DB state. The HB-to-DB transition caused by the kick is evident from the sharp change in H-bond population of the H-bond between the involved water molecules (O* and O) at *t* = 0 ps, which occurs *after* the abrupt increase of the O*–O distance initiated at earlier times *t* < 0 ps as highlighted in yellow in Figure 3(a). We note that after a sufficiently long time (around 1 ps) following the kick, the solvation shell water molecule eventually leaves the solvation shell of O*. Whereas, in bulk, almost all H-bond breaking events immediately cause the water molecule to escape from the solvation shell without any additional kick. Using again the CTRW model, diffusion due to these kicks can be calculated from *D*_kick_ = 2.5 × δ_kick_^2^/4τ_kick_, where the factor 2.5 appears due to the presence of neighboring HB pairs in a solvation basin, obtained from H-bond population in Figure 1(a); the corresponding time scale (τ_kick_) and displacement amplitude (δ_kick_) are enlisted in Table S1. The resulting value, *D*_kick_ ≈ 4.31 × 10^–5^ cm^2^s^–1^ (or *D*_frame_ ≈ 4.06 × 10^–5^ cm^2^s^–1^), together with *D*_SWE_ ≈ 1.45 × 10^–5^ cm^2^s^–1^, is found to *quantitatively* reproduce the overall diffusion ≈5.62 × 10^–5^ cm^2^s^–1^ (i.e., *D* ≈ *D*_SWE_ + *D*_kick_). Thus, *D*_kick_ and therefore kick events are found to contribute predominantly toward the overall diffusion of water in the confined system in stark contrast to what governs diffusion in bulk water.

**Figure 3:**
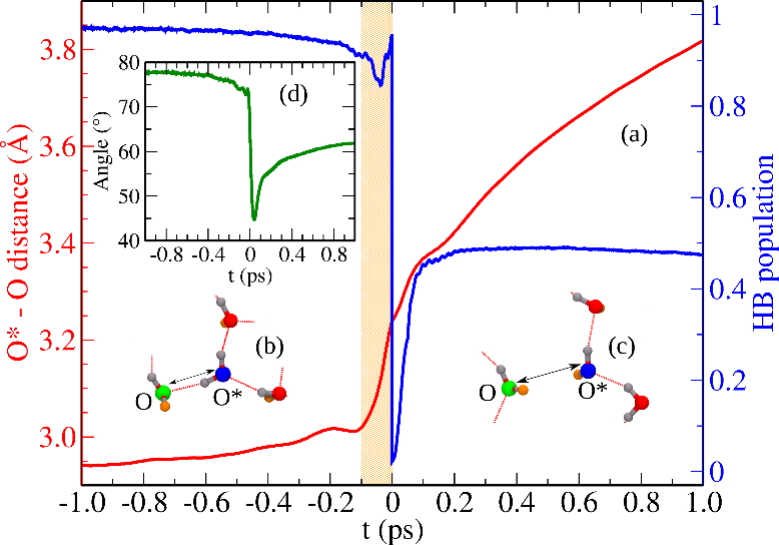
Change of (a) oxygen–oxygen distance (red, left scale), H-bond population (blue, right scale) and (d) angle between O–H and the surface normal with respect to the confining graphene walls during kick events, see the text for details; the yellow area marks the time regime *t* < 0 ps where the O*–O distance suddenly increases drastically while the H-bond population is not yet decreasing which only happens at *t* = 0 ps thus indicating kick-induced H-bond breaking between O and O* (recall that the shown functions have been averaged over very many individual kick events, see Supplemental Sec. 9). Panels (b) and (c) depict representative snapshots for the HB and DB states of the involved O–H bond associated with the central oxygen (shown in blue), respectively, wherein the significantly changing O*–O distance is marked by arrows.

This preceding analysis shows that the kick mechanism unveiled here explains why frame diffusion plays a most crucial role for the ultrafast dynamics of monolayer water at extreme confinement in slit pores. This is a completely distinct scenario from diffusion in bulk as well as interfacial water, where the translational dynamics is governed by the solvation water exchange mechanism.^34^

The diffusion of water molecules in monolayer lamellae within graphene-based slit pore setups is found to be ultrafast. The present study reveals that the mechanism behind this ultrafast diffusion is completely different from that in both, bulk and interfacial water. The key point here is that the geometric restriction due to such extreme confinement enforces a significant population of free or dangling O–H bonds. These bonds are not involved in H-bonding since they point toward the two walls. This in turn generates a highly frustrated H-bond network topology within the monolayer–absent in bulk and interfacial water. Transitions from H-bonded to dangling states are found to produce frequent kicks to the central water molecule while keeping its solvation shell intact, which in turn leads to ultrafast diffusion, even before exchanging or replacing any H-bonded partner. Such high mobility within a *persisting* solvation shell arrangement is an entirely new realization of H-bond dynamics found here to be the mechanistic cause of ultrafast diffusion of monolayer water in hydrophobic slit pores. This is in stark contrast to bulk and interfacial water where it is the solvation water *exchanges* that are essential for the diffusion process. Our stable state method utilizing solvation shell water exchanges is robust and general, thus broadly applicable to reveal different diffusion mechanisms, irrespective of the specific nature of the H-bond network and wall material. Thus, the current findings certainly provide a fundamental step toward understanding the peculiar dynamics of strongly confined aqueous solutions in more complex situations such as stratified ultranarrow biological compartments, Janus-type slit pores engineered by hydrophobic/hydrophilic walls in nanofluidic devices, modern electrochemical energy storage using 2D materials or layered minerals found within Earth’s crust.

## Supporting Information Available

Details of our simulation protocol, radial distribution functions, H-bond characteristics, solvation water exchange and kick mechanism, displacement amplitude and transition time for an event, calculation of diffusion coefficient from mean square displacement, statistical quality of transition properties, orientational and dipole relaxation, comparison of monolayer water with bulk and interfacial water (PDF)
