# Supplementary material for: Topological Frustration Triggers Ultrafast Dynamics of Monolayer Water Confined in Graphene Slit Pores
Source: Nano Lett. 2024 Nov 26;24(49):15623–8. doi: 10.1021/acs.nanolett.4c04077 (PMC11638954; doi:10.1021/acs.nanolett.4c04077)
Supplement: Supplementary file 1 — nl4c04077_si_001.pdf [file nl4c04077_si_001.pdf]

Supplemental Information to

Topological Frustration Triggers Ultrafast Dynamics of Monolayer Water Confined in Graphene Slit Pores

Banshi Das,[*,†] Sergi Ruiz-Barragan,[†,‡] Biman Bagchi,[†,¶] and Dominik Marx[†]

Lehrstuhl für Theoretische Chemie, Ruhr-Universität Bochum, 44780 Bochum, Germany

E-mail: banshi.das@rub.de

*To whom correspondence should be addressed

†Lehrstuhl für Theoretische Chemie, Ruhr-Universität Bochum, 44780 Bochum, Germany

‡Present address: Departament de Fisica, Universitat Politecnica de Catalunya, Rambla Sant Nebridi 22, 08222 Terrassa, Barcelona, Spain

¶Present address: Solid State and Structural Chemistry Unit, Indian Institute of Science, Bangalore 560012, Karnataka, India

Contents

1 Simulation Details **S3**

2 Radial Distribution Functions **S5**

3 H-bond Characteristics **S6**

4 Solvation Water Exchange **S8**

5 Kick Mechanism in Confined Monolayer **S10**

6 Displacement Amplitude **S12**

7 Transition Times **S13**

8 Mean Square Displacement **S14**

9 Statistical Quality of Transition Properties **S16**

10 Orientational and Dipole relaxation **S19**

11 Comparison of Monolayer Water with Bulk and Interfacial Water **S20**

1 Simulation Details

To simulate monolayer water in a slit pore setup based on two graphene walls, *ab initio* molecular dynamics[1] simulations have been carried out within a tailor-made QM/MM approach[1] where the interaction between water (QM subsystem) and graphene (MM subsystem) is described using a consistent interaction potential that has been optimized and validated previously.[2] Our QM/MM approach can efficiently and accurately generate extensive sampling trajectories, which would otherwise be computationally prohibitive with all-QM methods due to the additional presence of the graphene sheets. Additionally, it offers direct access to the full electronic structure information on-the-fly unlike force field simulations. This is required in the present study to compute dipole relaxation functions which we obtain from the effective electronic dipole moments of all water molecules based as usual on the maximally localized Wannier functions[1] assigned to the individual water molecules. The two perfectly coplanar graphene sheets contain 112 carbon atoms each and have been frozen at an average interlayer distance of $d_{\text{int}} = 6.68$ Å obtained from previous constant normal pressure ("rigid piston") simulations subject to the intrinsic density correction as introduced and validated earlier;[2] all details are provided in Section II of the corresponding Electronic Supporting Information. An earlier study[3] revealed the negligible impact of graphene sheet flexibility, as reflected in the nearly identical density and dielectric profiles compared to that of rigid and frozen sheets. Furthermore, a distinct VSFG spectrum at the limit of monolayer confinement, calculated using a similar frozen piston simulation,[4] closely aligns with a recent experiment.[5] In addition, we computed THz spectra of confined water between frozen graphene sheets and compared those to experimental such spectra[2] all providing further confirmation and validation of our methodology in comparison to intramolecular and intermolecular spectroscopy in the mid–IR and far–IR spectral regimes, respectively. The dimensions of the simulation box in the x, y, and z directions are 17.12, 17.30, and 21.68 Å, respectively, with each graphene sheet spanning the xy-plane. Here, we effectively decouple the interactions between periodic images in the z-direction by using a vacuum region along that direction,

as validated in previous studies by others and ourselves.[2,3,6,7] For the bulk water reference simulations, 128 water molecules are hosted in a periodic cubic supercell with a boxlength of 15.66 Å that establishes the experimental density at 300 K and 1 bar. The electronic structure of water is described using the RPBE-D3 density functional[8–10] in conjunction with GTH pseudopotentials,[11–13] TZV2P basis sets and a density cutoff of 500 Ry. For both systems, a long NVT trajectory at 300 K is generated to allow for sampling a set of initial conditions for the subsequent NVE simulations that are required to rigorously compute time-correlation functions in the canonical ensemble from the corresponding averages. This well-established approach allows us to rigorously calculate time-dependent properties of canonically distributed samples, such as time-correlation functions, while circumventing any possible dynamical artifacts caused by thermostatting. Using the two sets of initial phase space points from the NVT simulations, 60 independent microcanonical trajectories (each of 20 ps length) have been generated and used to carry out all structural and dynamical analyses presented herein. Using the protocol similar to that for the monolayer system,[2] we generated 12 sets of 20 ps long NVE trajectories for a wider gap (d_{int} = 14.69 Å) between the slit pores, with 105 water molecules in between. The simulation box dimensions in the x, y, and z directions are 17.12, 17.30, and 29.69 Å, respectively. At this wider pore limit, the interfacial layer behaves like a typical bare graphene-water interface.[2–4] The dynamical results for the interfacial water are compared with those of water at monolayer confinement in a subsequent section. We also produced 20 sets of 100 ps long NVE trajectories of bulk TIP4P/2005 water[14] using the same protocol to assess the time-dependence of the mean-square displacement used to compute diffusion coefficients as discussed later. All simulations have been performed with `CP2k` simulation package.[15–18]

2 Radial Distribution Functions

The radial distribution function (RDF), which captures the structural correlation due to solvation, is expressed in general as

$$g(r) = \frac{N(r)}{V(r) \times \rho} \tag{1}$$

where $N(r)$ is the number of species within a shell between r and $r + \mathrm{dr}$ around a similar or different species present in the system, $V(r)$ is volume of that shell and ρ is the (bulk) density of that species in the system. For bulk system, the volume is simply given by $V(r) = 4\pi r^2 \mathrm{d}r$, being the volume of spherical shell of radius r. The same volume element should not be used for the confined monolayer due to its anisotropic nature along the surface normal (here the z-direction). Here, instead of the full spherical volume, due to the slab geometry of the confined monolayer, we have used a truncated version[19] given by $V(r) = 2\pi d_{\mathrm{wat}} r \mathrm{d}r$, where d_{wat} is obtained from the interlayer distance d_{int} defined above from which the van der Waals diameter of carbon ($d_{\mathrm{vdW}} = 3.40$ Å) has been subtracted, thus providing the accessible width available for water molecules along the z-direction. Similarly for the density (ρ) calculation, the total volume for the confined monolayer water is taken as (x-dimension)$\times$(y-dimension)$\times$(d_{wat}). The free energetics of the solvation can be understood in terms of the potential of mean force, $W(r) = -k_{\mathrm{B}}T \ln g(r)$.

From the comparison of the oxygen-oxygen RDF as depicted in Fig. S1(a), the first solvation shell is found to be more pronounced for the monolayer water due to the increase in density within the restricted slit pore geometry. The second shell is much broader and appears at much larger distance for the confined monolayer water as the H-bond network topology is frustrated due to the presence of many O-H dangling bonds that point out of the thin layer toward the two nearby graphene walls, whereas the unperturbed three-dimensional H-bond network is established in bulk water. This is the reason behind the larger separation of a water molecule after escaping from a solvation shell in monolayer confinement as discussed in the main text, based on Fig. 2(a) therein, compared to what is known for bulk water. The extended solvation shell width together with the significantly

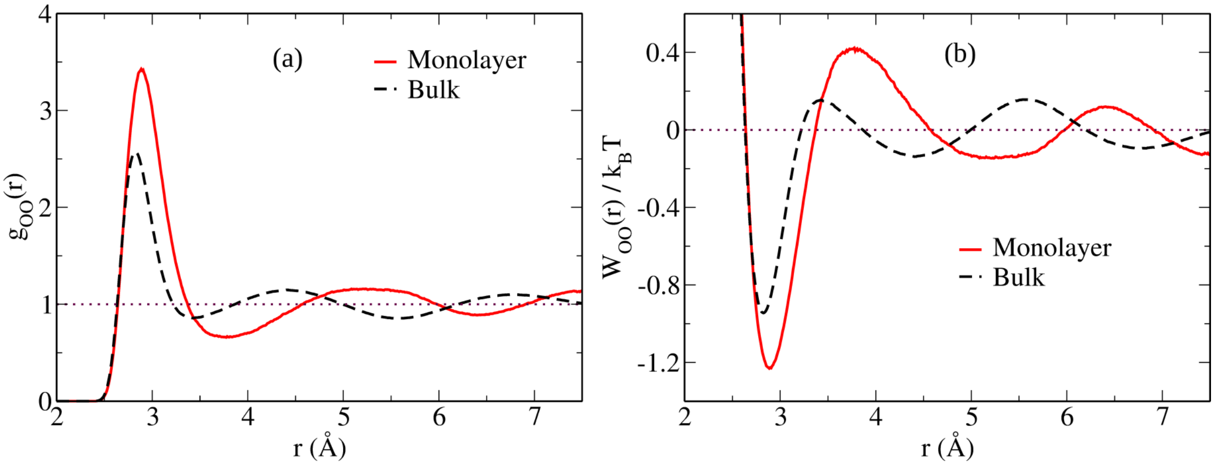

Figure S1: (a) Radial distribution function and (b) potential of mean force of oxygen-oxygen pairs in confined monolayer and bulk water; the horizontal dotted lines guide the eye.

higher activation free energy to escape from the solvation shell as depicted in Fig. S1(b) is the reason for the much less frequent "solvation water exchange" (SWE) events (see τ_{SWE} in Sec. 7 herein) despite of the much more labile nature of H-bonds in the monolayer (shown in Fig. 1(b) in the main text).

3 H-bond Characteristics

To identify H-bonds between donor-acceptor pairs, we calculated the joint probability distributions of intermolecular O$\cdots$H distances and O–H$\cdots$O angles. As depicted in Fig. S2, the H-bonded region (in the lower-left corner) for both, monolayer and bulk water is described by the same separatrix given by $r_{\mathrm{O}\cdots\mathrm{H}} < -1.71\cos(\theta_{\mathrm{O-H}\cdots\mathrm{O}}) + 1.37$, which we thus use to identify (tag) H-bonds in the monolayer as well as in the bulk system. The structural relaxation of H-bond networks is usually quantified using the intermittent H-bond correlation function, $C(t) = \langle h(0)h(t)\rangle/\langle h\rangle$, where $h(t) = 1$ when an H-bond exists between a pair of water molecules at time t and otherwise it is zero. We have calculated this H-bond correlation function for H-bonds between donor-acceptor pairs ($C_{\mathrm{DA}}(t)$) and also for water-water pairs ($C_{\mathrm{WW}}(t)$) irrespective of the specific donor-acceptor combination (Fig. S3). The timescales for the corresponding H-bond relaxation

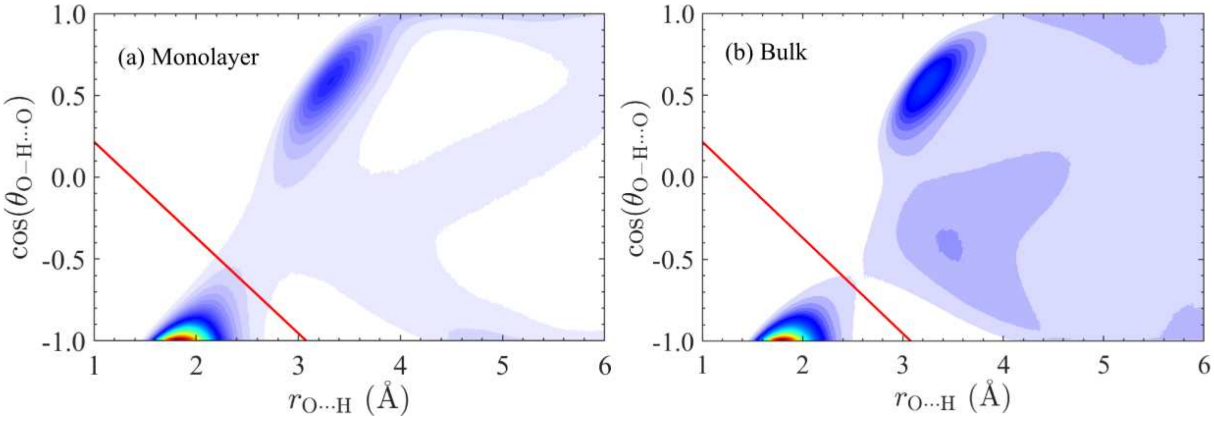

Figure S2: Joint probability distribution functions of intermolecular O$\cdots$H distances and $\theta_{O-H\cdots O}$ angles for (a) confined monolayer and (b) bulk water. The area in the lower left corner separated by the solid red line (see text for parameterization) provides the region where H-bonds exist between two water molecules.

is calculated from the integration after bi-exponential fitting, yielding for monolayer water $\tau_{DA} = 2.19$ ps and $\tau_{WW} = 6.44$ ps while bulk water is characterized by $\tau_{DA} = 4.16$ ps and $\tau_{WW} = 6.46$ ps. Thus, a significant slowing-down ratio of water-water H-bond relaxation is found in the confined monolayer ($\tau_{WW}/\tau_{DA} = 2.94$) as compared to bulk ($\tau_{WW}/\tau_{DA} = 1.55$). The enormous factor-of-two difference between the two systems indicates that

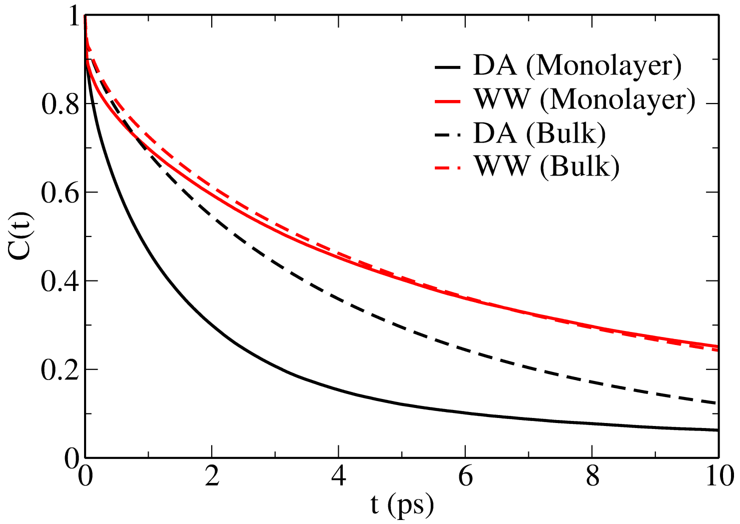

Figure S3: Intermittent H-bond correlation functions between donor-acceptor pairs, $C_{DA}(t)$, and water-water pairs, $C_{WW}(t)$, as defined in the text for confined monolayer and bulk water.

although H-bonds between a particular donor-acceptor pair break much more frequently

in the monolayer, there is a higher probability to reform that H-bond within the same water pair – albeit with a different donor-acceptor combination. This is traced back to the presence of many dangling O–H bonds in the confined monolayer, which triggers the frequent breaking of H-bonds between a specific donor-acceptor pair but instead of leaving the H-bond shell (as observed in bulk water), a H-bond between the same water pair is reformed subject to a different donor-acceptor combination.

4 Solvation Water Exchange

Based on the obtained understanding of the flexible nature of H-bonds in monolayer water as presented in the main text, we now propose to use a stable state description in terms of the entire solvation shell (thus introducing "solvation water exchange" (SWE) events and τ_{SWE}, see below) instead of using H-bonded pairs to define stable states as introduced earlier.[20–22] We first select all coordinated waters around a central water molecule based on their O–O distances: If the O–O distance is less than the distance corresponding to the first minimum of the respective radial distribution function ($\approx$ 3.75 Å for monolayer and 3.40 Å for bulk water), we consider that water molecule as a member of the coordination shell. The resulting probability distributions of the coordination number (Fig. S4(a))

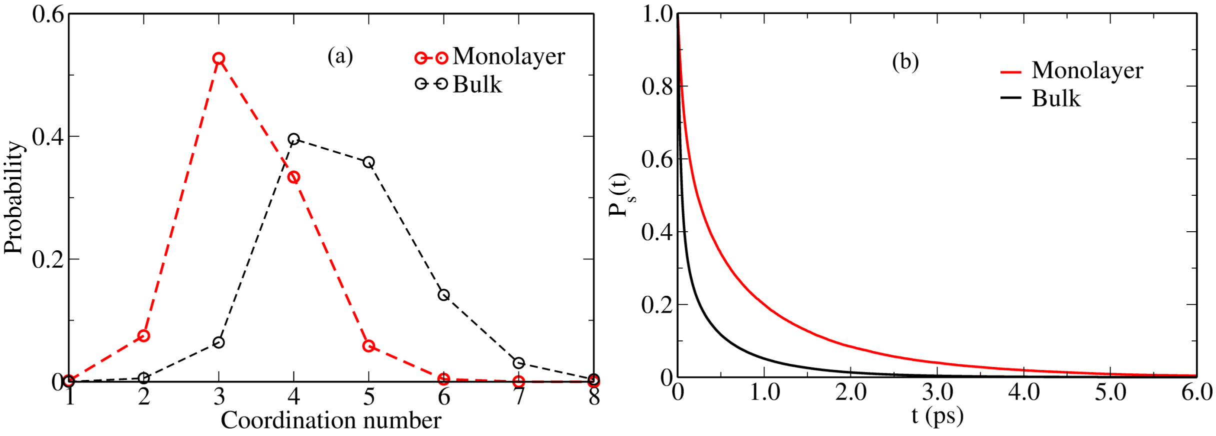

Figure S4: (a) Probability distributions of coordination number and (b) survival probabilities of a stable state defined in terms of solvation shell for confined monolayer (red) and bulk (black) water.

clearly capture the different most probable states, which corresponds to a three-fold (or

four-fold) coordinated solvation shell for monolayer (or bulk) water. We now consider these three (or four) coordinated solvation shells as the respective stable state for the monolayer (or bulk) water, and all other states are then considered as local fluctuations around the respective stable state. We first calculate the survival probability of a three (or four) coordinated state as, $P_s(t) = 1 - \int_0^t \mathrm{d}t'\, \psi(t')$, where $\psi(t')$ is the waiting time of a particular state (see Fig. S4(b) for the normalized survival probability). The two timescales for the decay of the survival probability are extracted from bi-exponential fitting as usual. The fast timescale (0.12 ps for monolayer and 0.05 ps for bulk water) in the decay of $P_s(t)$ appears due to the transient leaving from a stable state, which is considered here as a fluctuation within the *same stable state.* The second timescale (1.12 ps for monolayer and 0.56 ps for bulk water), however, captures the escape from one stable state to a another stable state. We now identify all productive transition events by not considering the fluctuations within a stable state which occur on ultrafast timescales (of less than 0.12 ps for monolayer and 0.05 ps for bulk water). The transitions between these stable states are found to be associated with the exchange of a solvation shell water molecule with another molecule from outside the solvation shell, termed here "solvation water exchange" (SWE) events, which is then traced in time using their distances from the central water molecule in the solvation shell as described in the main text and analyzed in Fig. 2 therein.

It should be noted that our solvation water exchange picture is different from the H-bonded pair exchange[22] commonly used for bulk as well as interfaces. Considering the bulk limit, a water molecule has mostly four H-bonded partners around it. Any such HB partner can get exchanged with another water molecule via a large-amplitude jump. The contribution from each such HB exchange event can then be quantified using the CTRW model[22,23] based on $\delta^2_{\mathrm{O}*}/6\tau_{\mathrm{HB}}$. As there are mostly four such HB partners around a central water molecule, the total contribution from all HB partners due to H-bonded pair exchange would be $4 \times \delta^2_{\mathrm{O}*}/6\tau_{\mathrm{HB}}$. Now, in the SWE picture, we consider the whole solvation shell (not H-bonded pairs anymore). If any of the mostly four solvation water molecules in the bulk get exchanged we call it a SWE event. As any of the four HB partners within the solvation shell can be replaced in the SWE process, the rate of the

SWE is roughly four times that of the HB exchange events, ($1/\tau_{\mathrm{SWE}} \approx 4 \times 1/\tau_{\mathrm{HB}}$). Thus, the diffusion contribution due to solvation water exchange is about $\delta^2_{\mathrm{O}*}/6\tau_{\mathrm{SWE}}$, which is $\approx 4 \times \delta^2_{\mathrm{O}*}/6\tau_{\mathrm{HB}}$, thus capturing the same diffusion process as the one described in earlier analysis of bulk water based on H-bonded pair exchange events,[22] but using in this work a different representation and thus computational formalism.

5 Kick Mechanism in Confined Monolayer

In the confined monolayer, when a solvation shell water breaks a H-bond with the central water molecule, it imparts a kick to the central water molecule. In this process, one

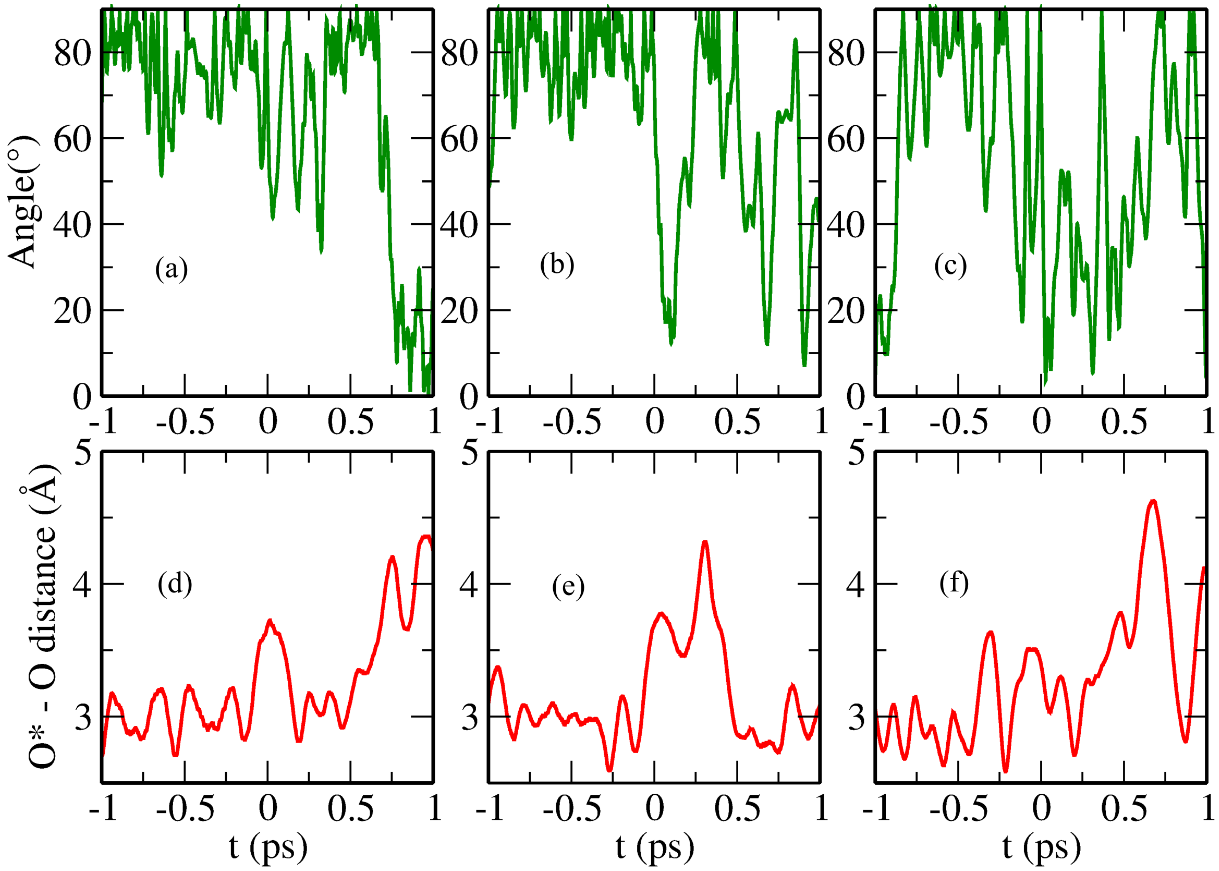

Figure S5: Variations of O–H angles with respect to the surface normal (panels (a) to (c)) and O–O distances (panels (d) to (f)) for three representative individual kick events.

O-H bond transition from an HB to a DB (dangling bond) state leads to an increased separation between the solvation shell water molecule and the central water molecule.

Instead of leaving the solvation shell, the water molecule can approach and re-establish a hydrogen bond with the central water molecule. In Fig. S5, we depict three representative trajectory fragments where the water molecules are considerably separated, making the existing hydrogen bond unsustainable. Nonetheless, rather than leaving the solvation shell, they approach each other to re-establish a H-bond again. The orientation of the associated O-H bond is found to change during what we call "kick events". Before the kick (when $t < 0$), the associated O-H bond is in a HB state, hence mostly parallel to the confining walls. After transition to the DB state, that O-H bond inclines towards the walls and the angle between the O-H bond and surface normal decreases (Fig. S5). As water molecules weakly interact with the graphene sheets, the angle in the DB state fluctuates within a considerable range of about 10 to 60° compared to that in the HB state (Fig. S6). In Fig. 3 of the main text, the distance and angle variations are depicted after averaging over all such events, while ignoring the transient breaking and reformation of H-bonds occurring on an ultrafast timescale (here 0.05 ps).

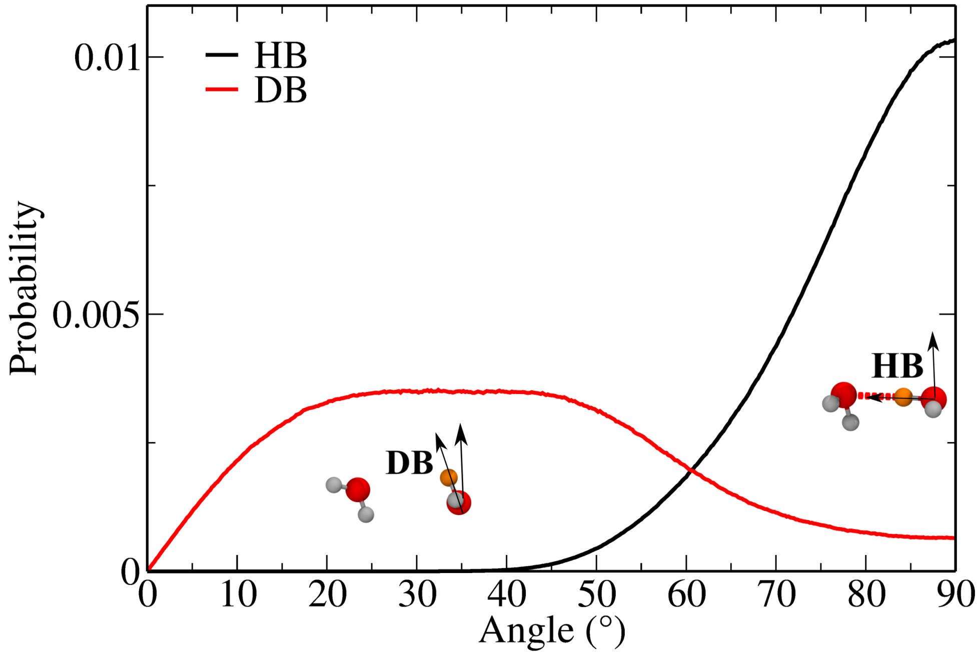

Figure S6: Probability distributions of O–H angles with respect to the surface normal for HB and DB states, see text. The representative snapshots characterizing the HB and DB states are shown in the insets, where the vertical arrows correspond to the surface normal vector while the other corresponds to the direction of the O-H bond considered.

6 Displacement Amplitude

In order to calculate the displacement amplitude associated with solvation water exchange (SWE) or kick events, we need to identify the time span of the corresponding structural transition, which can be considered as the time window of the first appearance of the stable state before and after the transition state. Following earlier work,[22] we calculate the velocity autocorrelation function of oxygen atoms with respect to the time origin of the particular event as $C_{\mathrm{vv}}(t) = \langle \vec{v}_{\mathrm{O}}(t_0) \cdot \vec{v}_{\mathrm{O}}(t_0 + t)\rangle / \langle \vec{v}^2_{\mathrm{O}}(t_0)\rangle$, where the first minimum of $C_{\mathrm{vv}}(t)$ corresponds to the appearance of the stable state while crossing the transition state. As water molecules in the confined monolayer are restricted to move parallel to two

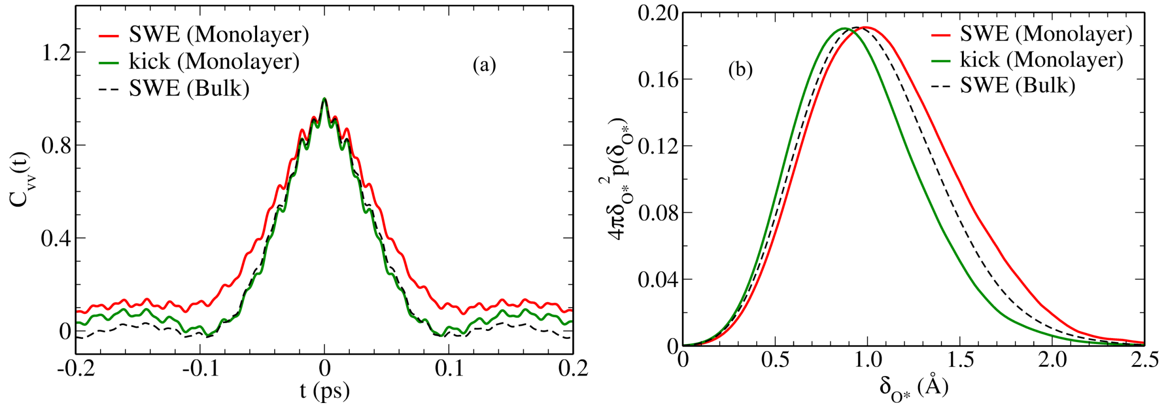

Figure S7: (a) Velocity autocorrelation function of the oxygen atom (O*) of the central (reference) water molecule with respect to the time origin of solvation water exchange (SWE) and kick events, see text, and (b) jump amplitude probability density for the corresponding transitions; note that kick events are absent in bulk water as elaborated in the main text.

graphene sheets, i.e. essentially in the xy-plane, we consider only the xy-component of the velocity in the calculation of this autocorrelation function. We find that the velocity correlation of the central water molecule exhibits first minima around time differences Δt of 0.1 ps in both the positive and negative time with respect to the time origin for both, SWE as well as kick events (Fig. S7(a)). Hence, the spatial displacement amplitude for a transition can be calculated as[22] $\vec{\delta}_{\mathrm{O}*} = \vec{r}_{\mathrm{O}*}(t_0 + \Delta t) - \vec{r}_{\mathrm{O}*}(t_0 - \Delta t)$, where $\Delta t = 0.1$ ps and t_0 is the time origin of the considered events. The probability distribution of these displacement amplitudes averaged over all observed events are depicted in Fig. S7(b) and

the mean of the squared amplitudes, $\delta^2_{\mathrm{O}*}$, are reported in Table S1.

Table S1: Average squared amplitude of displacements ($\delta^2_{\mathrm{O}*}$) and transition times (τ) for solvation water exchange (SWE) and kick events as described in the text for confined monolayer and bulk water; note that kick events are absent in bulk water as elaborated in the main text.

System	$\delta^2_{\mathrm{O}*}$ (SWE)	τ_{SWE}	$\delta^2_{\mathrm{O}*}$ (kick)	τ_{kick}
Monolayer	0.76 Å^2	1.31 ps	0.58 Å^2	0.84 ps
Bulk	0.64 Å^2	0.75 ps	–	–

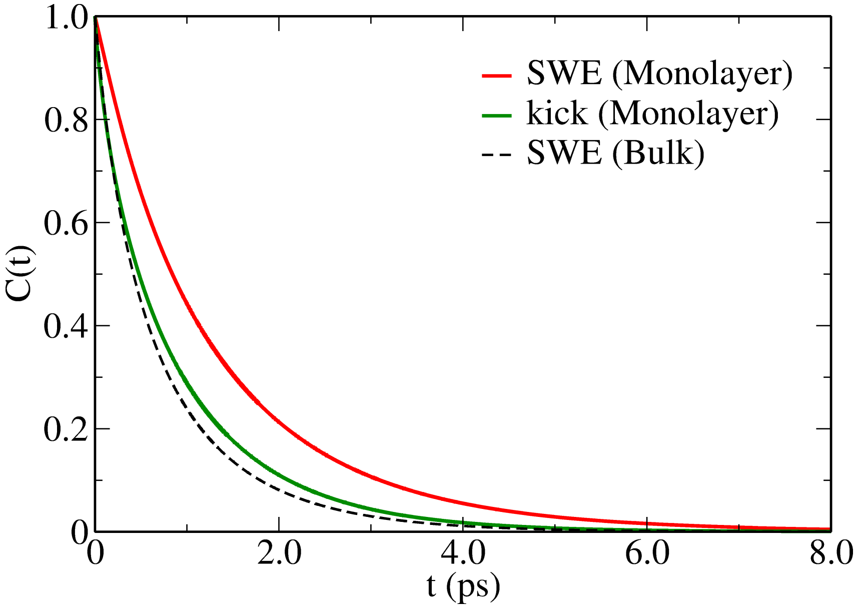

Figure S8: Transition (jump) time correlation functions for solvation water exchange (SWE) and kick events as described in the text for confined monolayer and bulk water; note that kick events are absent in bulk water as elaborated in the main text.

7 Transition Times

The time required for a transition from an initial (i) to a final state (f) in either solvation water exchange (SWE) or kick events can be calculated as[22]

$$\tau = \int_0^\infty \mathrm{d}t \left[1 - \langle p_{\mathrm{i}}(0) p_{\mathrm{f}}(t) \rangle\right], \tag{2}$$

where the population of the initial state p_i (or p_f for the final state) is 1 before (or after) the event and zero otherwise. The corresponding transition (jump) time correlation functions ($[1 - \langle p_\mathrm{i}(0)p_\mathrm{f}(t)\rangle]$) for the SWE/kick events are depicted in Fig. S8 and the corresponding integrated timescales τ are compiled in Table S1; note that kick events are absent in bulk water as elaborated in the main text.

8 Mean Square Displacement

Diffusion coefficient is generally obtained from the mean-square displacement (MSD) of a molecule, calculated from a MD trajectory. In the diffusive regime, MSD is linear and the slope is related to the self-diffusion coefficient (D), which is $6\times D$ for bulk and $4\times D$ for monolayer water. To unveil the underlying molecular mechanism of diffusion, we calculate the MSD of a water molecule residing in a stable state (frame contribution as described in main text) and the diffusion coefficient is then extracted from the linear fitting of the corresponding MSD in the rather small time window from 0.5 to 1.2 ps which is limited by the short transition time of the stable state. The overall diffusion coefficient for bulk and monolayer water is obtained as 1.88 and 5.62 $\times$ 10^{-5} cm^{2}s^{-1}, respectively (Fig. S9(a)).

To check the effect of the time window on the computed diffusion coefficients, we also extract the (overall) diffusion coefficient from the linear fit in a time window from 2 to 10 ps as shown in the inset of Fig. S9(a). Importantly, for this larger time window, the diffusion coefficients are found to be very similar (namely 1.78 for bulk and 5.26 $\times$ 10^{-5} cm^{2}s^{-1} for monolayer water) to those obtained using the smaller time window (0.5–1.2 ps) as discussed above. We also access a much longer time window (10–50 ps) using force field simulations of TIP4P/2005 bulk water based on the same sampling protocol as for the *ab initio* simulations used for all other reported analyses, where the diffusion coefficient (2.00 $\times$ 10^{-5} cm^{2}s^{-1}) aligns with the values of 1.98 and 2.10 $\times$ 10^{-5} cm^{2}s^{-1} obtained when fitting the MSD in the 2–10 and 0.5–1.2 ps time intervals, respectively. After this validation, the frame diffusion values, those are constrained by the small jump time, are calculated from the linear fit of the MSD within the smaller time window 0.5 to 1.2 ps (Fig. S9), and these values are 0.63 $\times$ 10^{-5} cm^{2}s^{-1} for bulk and 4.06 $\times$ 10^{-5} cm^{2}s^{-1} for

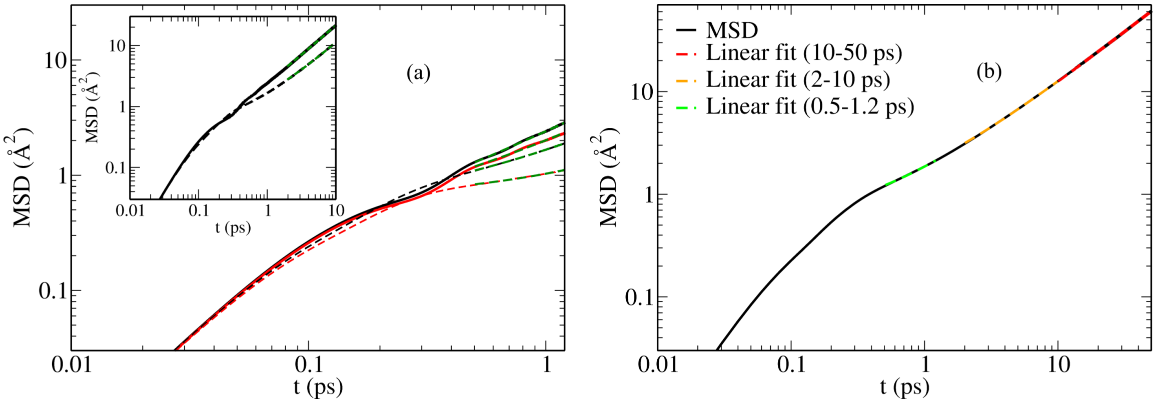

Figure S9: (a) Mean-square displacement (averaged over all water) of confined monolayer (black solid) and bulk (black dashed) water and MSD of water molecules in a H-bonded shell in confined monolayer (red solid) and bulk (red dashed) water. The linear fits (0.5 to 1.2 ps) are shown by green dashed lines. In the inset, MSD of monolayer (black solid) and bulk (black dashed) water are shown upto a longer time 10 ps and the linear fits (2 to 10 ps) are shown by green dashed lines. (b) MSD of TIP4P/2005 bulk water and its linear fits in three different time intervals. All linear fits to determine the self-diffusion coefficients result in a correlation coefficient (R) of 0.999. See text for discussion.

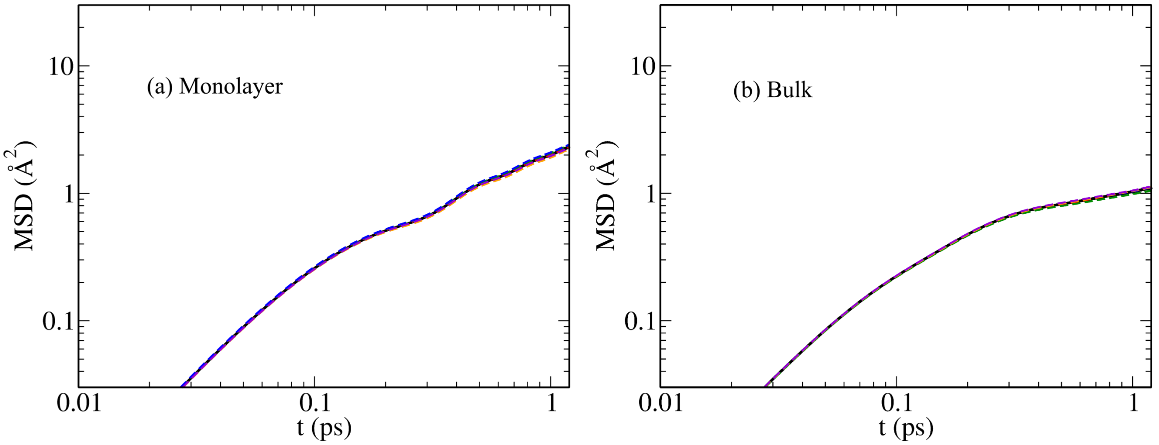

Figure S10: Mean-square displacement of the frame contribution averaged over all the trajectories (black solid lines) and 5 blocks (dashed) for (a) confined monolayer and (b) bulk water.

monolayer water.

To check the convergence of the frame diffusion as obtained from the present approach, we divided the total set of 60 trajectories into 5 blocks with 12 trajectories in each block and calculated the MSD of the stable state (providing the frame contribution) for all 5 blocks. As can be seen in Fig. S10, a well converged value of the frame diffusion coefficient can be obtained from our *ab initio* simulation data for monolayer (a) and bulk (b) water

even when using the short time window that is limited by the average transition time of the stable state. Notably, the bulk water reference value of 0.63×10^{-5} cm^{2}s^{-1} obtained with our procedure is in excellent agreement with the earlier reported value of 0.623×10^{-5} cm^{2}s^{-1} obtained from a constrained simulation of TIP4P/2005 bulk water.[22]

9 Statistical Quality of Transition Properties

The statistical quality of any property (x) associated with the transition events can be measured using the 95 % confidence interval as follows,

$$CI = \bar{x} \pm 1.96 \left(\frac{\sigma}{\sqrt{N}} \right), \tag{3}$$

where the average value of x is calculated as $\bar{x} = \frac{1}{N} \sum_{i=1}^{N} x_i$ and the standard deviation from $\sigma = \sqrt{\frac{1}{N} \sum_{i=1}^{N} (x_i - \bar{x})^2}$ for a number N of total events. For monolayer water, we find a total of 19055 SWE events and 99443 kick events, and for bulk water we identify a total of 205187 SWE events. Based on this significant statistics the error for all quantities are found to be negligibly small at the scale of the average values (see Fig. S11 for the SWE events and Fig. S12 for the kick events).

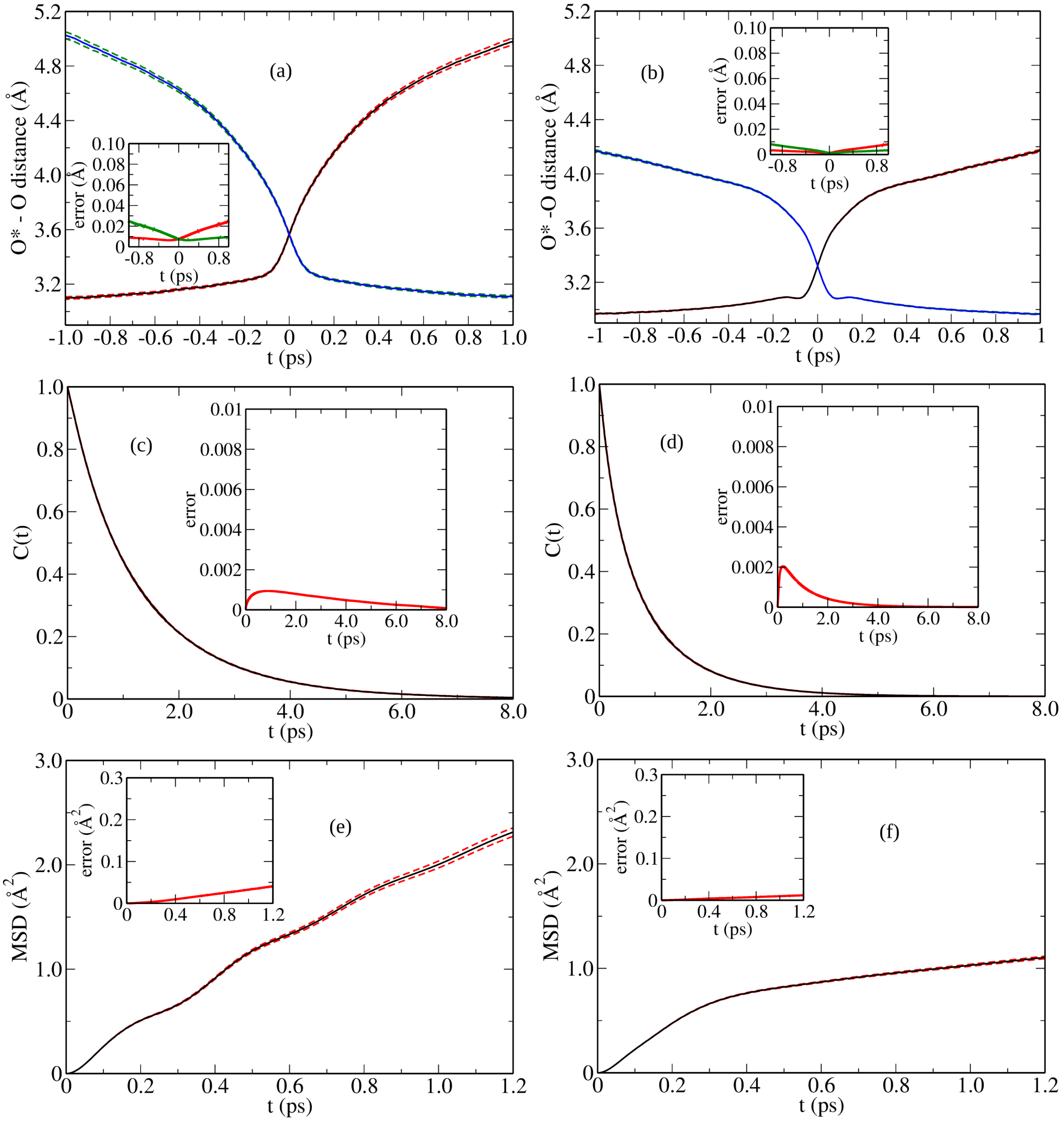

Figure S11: O*–Oa and O*–Ob distances during SWE events for (a) confined monolayer and (b) bulk water, transition time correlation function for SWE events for (c) confined monolayer and (d) bulk water, mean square displacement within a stable solvation shell for (e) confined monolayer and (f) bulk water, are shown using solid lines. The 95 % confidence interval is shown by dashed lines and the error estimate for each quantity is displayed in the inset of the respective figure.

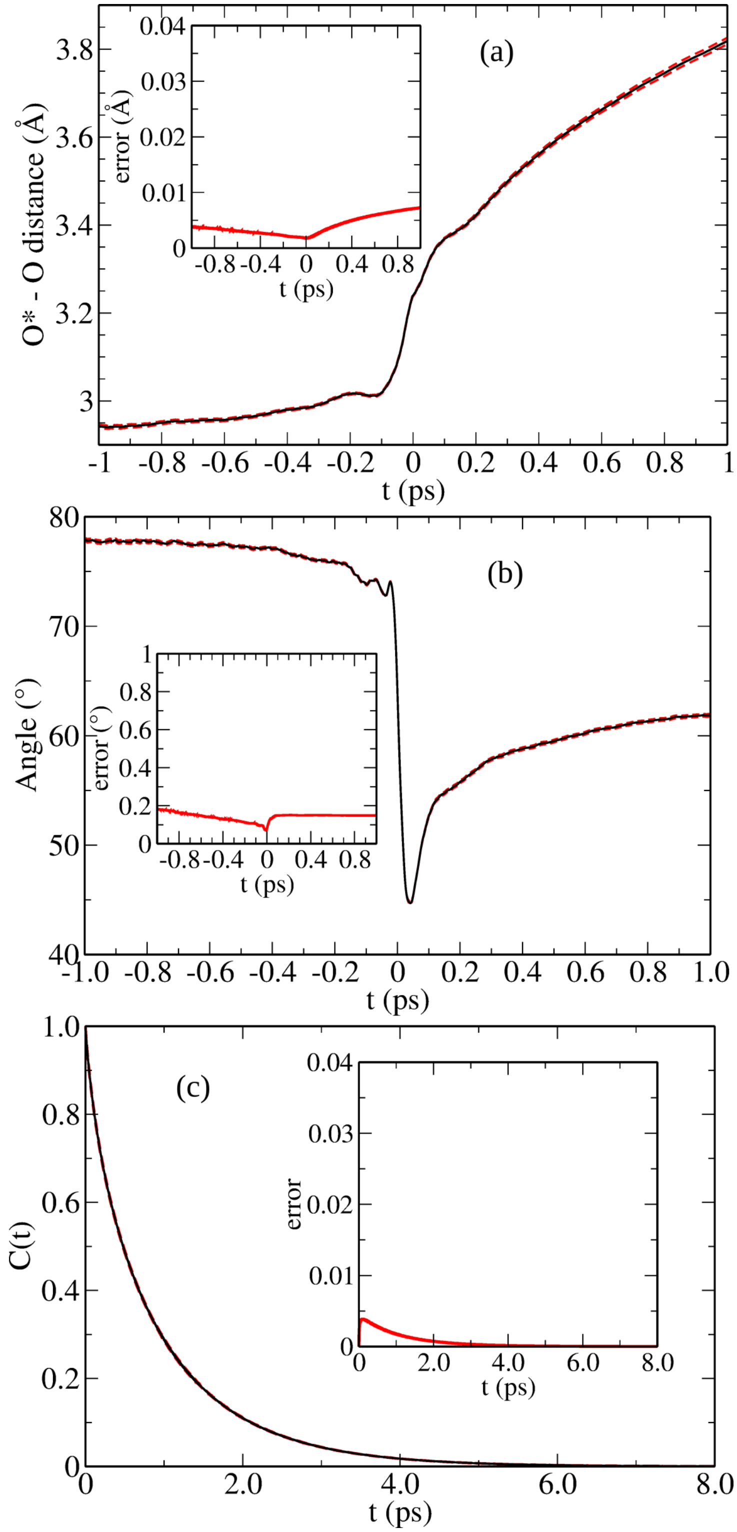

Figure S12: (a) O*–O distance, (b) angle between O–H and the surface normal with respect to the confining walls during kick events, and (c) transition time correlation function for kick events for confined monolayer water are shown using solid lines. The 95 % confidence interval is shown by dashed lines and the error estimate for each quantity is displayed in the inset of the respective figure.

10 Orientational and Dipole relaxation

In H-bond network systems like liquid water, H-bond breaking and reformation is associated with both translational and rotational dynamics of the water molecules. The rotational dynamics can be probed using anisotropic measurements based on polarization resolved pump probe experiments[24–26] and can be analyzed in terms of the orientational time correlation function of the O–H bond as follows,

$$C_l^{\mathrm{OH}}(t) = \frac{\langle P_l(e^{\mathrm{OH}}(t) \cdot e^{\mathrm{OH}}(0)) \rangle}{\langle P_l(e^{\mathrm{OH}}(0) \cdot e^{\mathrm{OH}}(0)) \rangle}, \quad (4)$$

where P_l is the Legendre polynomial of rank l and $e^{\mathrm{OH}}(t)$ is the unit vector along O–H direction at time t.

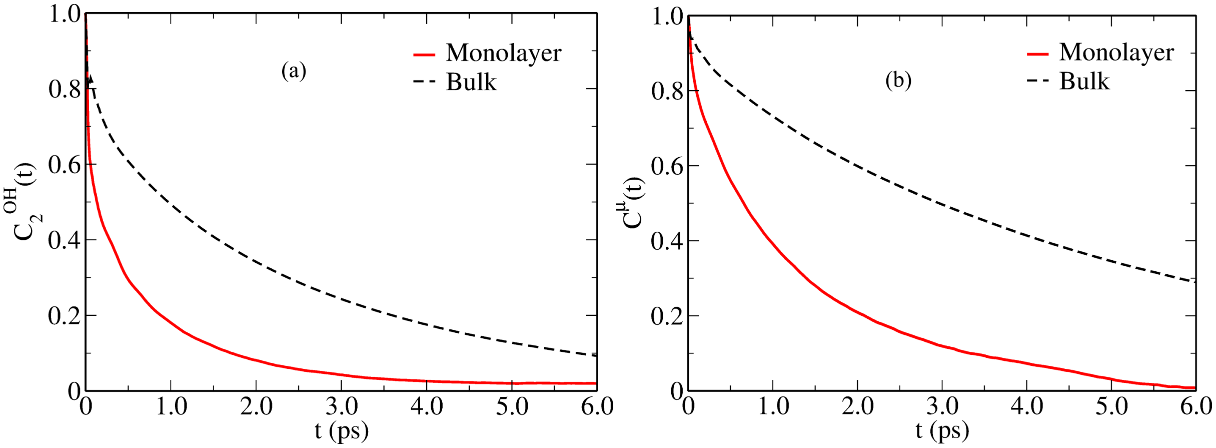

Figure S13: Relaxation of (a) 2nd rank orientational correlation function of the O-H vectors and (b) dipole-dipole correlation of the water molecules for confined monolayer and bulk water.

The timescales of the the 2nd rank ($l = 2$) orientational correlation extracted with the usual tri-exponential fitting are enlisted in Table S2. The slowest timescale (3.07 ps) for the bulk is consistent with the earlier observation[20,27] that the HB exchange (τ_{HB}) is associated with the reorientation of the O-H bond, which is here related to the solvation water exchange time ($4 \times \tau_{SWE}$). However for monolayer water the most important slow time component 0.80 ps is associated with the fast HB-to-DB transition time (τ_{kick}). An additional component with a very small weight (only 4 % of the total) is found

for monolayer water, as previously noted for water in hydrophobic confinement.[28] The relaxation of dipole-dipole correlation of the water molecules can be obtained as,

$$C^{\mu}(t) = \frac{\langle \mu(t) \cdot \mu(0) \rangle}{\langle \mu(0) \cdot \mu(0) \rangle}. \tag{5}$$

The effective molecular electronic dipole moment vector of a water molecule ($\mu(t)$) at time t can be calculated as $\sum_i^{N_{\text{nuclei}}} q_i r_i(t) - 2\sum_j^{N_{\text{wannier}}} r_j(t)$, where q_i and r_i are the charges and positions of the nuclei (O, H, H for water), and r_j are the positions of the maximally localized Wannier centers (as for instance explained in Ref. 1). The overall decay times are obtained from the integration of the tri-exponential fits of the correlation functions shown in Fig. S13(b). The dipole relaxation of water molecule is also found to be ultrafast for the monolayer confinement (1.22 ps) as compared to that of bulk (4.78 ps).

Table S2: Timescales of the decay of the 2nd rank O–H orientational correlation function for bulk and monolayer confined water; τ_1, τ_2, τ_3 (in ps) are extracted from the tri-exponential fitting with the weights of a_1, a_2, a_3 respectively, reported in parentheses.

System	τ_1 (a_1)	τ_2 (a_2)	τ_3 (a_3)
Monolayer	0.04 (0.46)	0.80 (0.50)	7.61 (0.04)
Bulk	0.01 (0.17)	0.43 (0.18)	3.07 (0.65)

11 Comparison of Monolayer Water with Bulk and Interfacial Water

For interfacial water, we select the respective water molecules from the interfacial layer adjacent to the graphene sheet in the slit pore simulation with a large pore size (d_{int} = 14.69 Å) as explained and validated above. Here we discuss the key quantities of interfacial water that influence its dynamics in comparison to both confined monolayer and bulk water.

The truncation of the liquid phase at the interface results in a higher number of dangling bonds compared to bulk water as indicated by the increased population of 3-

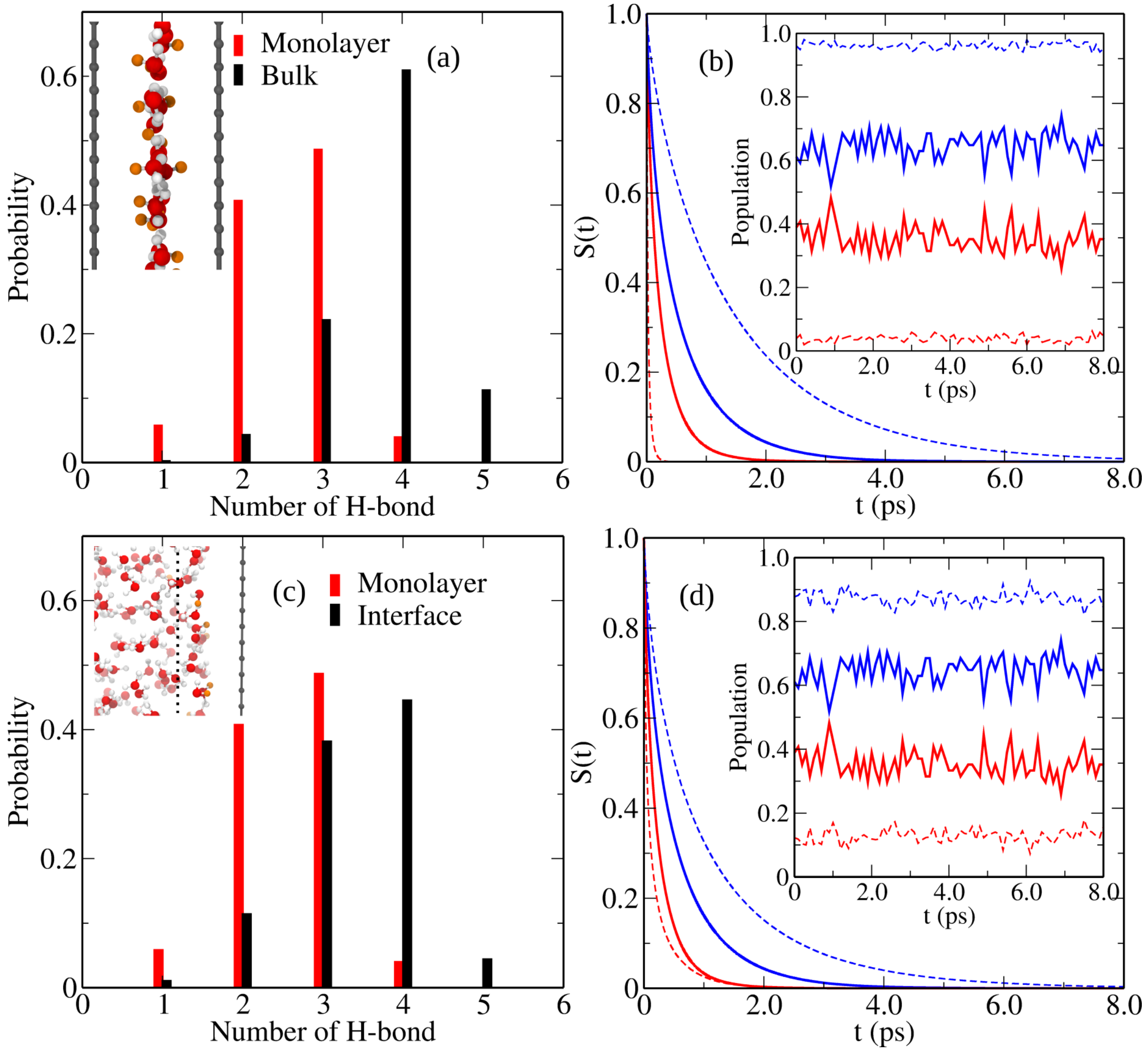

Figure S14: (a) Hydrogen bond populations in confined monolayer (red) and bulk (black) water; a representative snapshot of monolayer water confined between two graphene walls is shown in the inset. (b) Continuous time correlation functions of dangling bond (DB, red) and hydrogen bond (HB, blue) states for confined monolayer (solid) and bulk (dashed) water; the representative time evolution of the DB/HB populations is shown in the inset for a typical trajectory segment. (c) Hydrogen bond populations for confined monolayer (red) and interfacial (black) water; a representative snapshot of interfacial water adjacent to the graphene wall separated by a dotted line is shown in the inset. (d) Continuous time correlation functions of dangling bond (DB, red) and hydrogen bond (HB, blue) states for confined monolayer (solid) and interfacial (dashed) water; the representative time evolution of the DB/HB populations is shown in the inset for a typical trajectory segment.

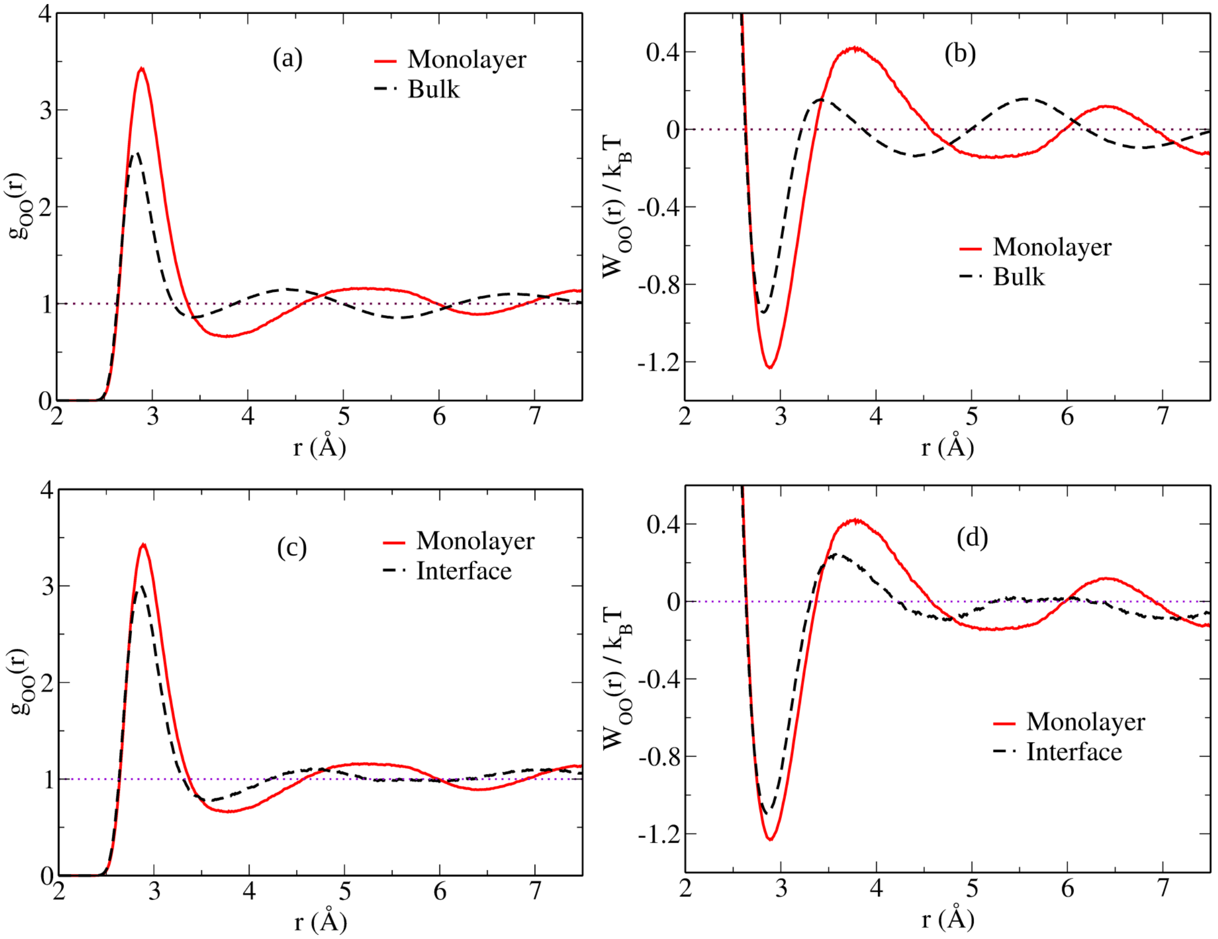

Figure S15: (a) Radial distribution function and (b) potential of mean force of oxygen-oxygen pairs in confined monolayer and bulk water. (c) Radial distribution function and (d) potential of mean force of oxygen-oxygen pairs for confined monolayer and interfacial water. The horizontal dotted lines guide the eye.

coordinated H-bonds at the interface (Fig. S14). However, unlike monolayer water, where geometric restriction by both sheets in the H-bond network allows only 2- or 3-coordinated H-bonds for a water molecule, the population of dangling bonds at the interface is higher than in the bulk but still much lower than in monolayer water. A significant number of dangling bonds are present at the interface, although this is much lower than in monolayer water (Fig. S14). These dangling bonds at the interface are not as transient as those in the bulk, with τ_{DB} being approximately six times faster than τ_{HB}, yet they are not as prominent as in monolayer confinement (Fig. S14).

The larger population of dangling bonds broadens the solvation shell compared to the bulk, though it remains narrower than in monolayer confinement (Fig. S15). The

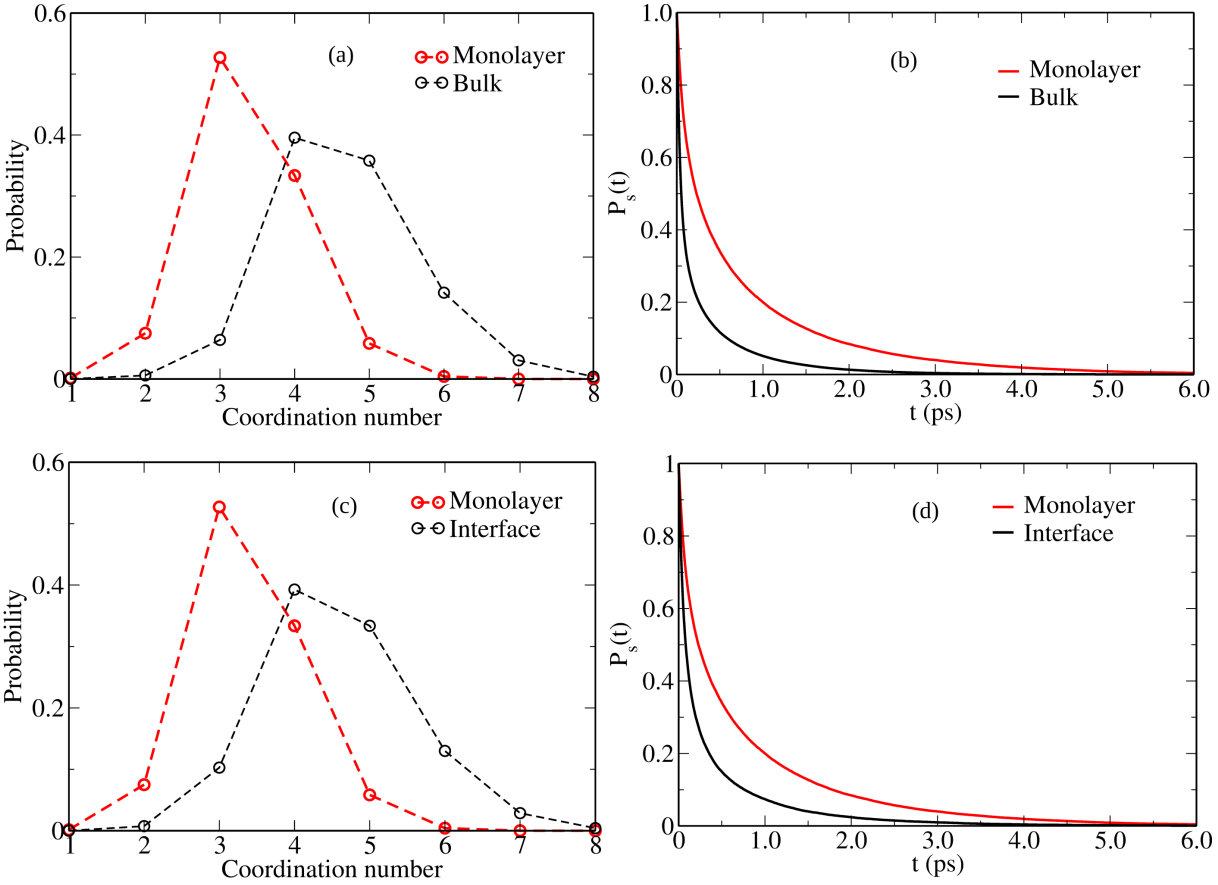

Figure S16: (a) Probability distributions of coordination numbers and (b) survival probabilities of a stable state defined in terms of the solvation shell for confined monolayer (red) and bulk (black) water. (c) Probability distributions of coordination numbers and (d) survival probabilities of a stable state defined in terms of the solvation shell for confined monolayer (red) and interfacial (black) water.

overall 4-coordination of the solvation shell makes the stable state at the interface more akin to the bulk than to monolayer water (Fig. S16). Using the protocol described in Sec. 4, we identify all solvation water exchange (SWE) events. Similar to monolayer water, the O*–Oa (or O*–Ob) separation distance is greater before (or after) the exchange event (Fig. S17), which results from the flexible nature of the broader solvation shell of interfacial water compared to that of the bulk. The diffusion coefficient of interfacial water, determined using the xy-component of the mean square displacement (Fig. S19), is found to be 2.32×10^{-5} $\mathrm{cm^2 s^{-1}}$. The frame diffusion coefficient extracted from the xy-component of the mean square displacement (Fig. S19) of water molecules within a specific solvation shell at the interface is found to be 0.98×10^{-5} $\mathrm{cm^2 s^{-1}}$, which is higher than that

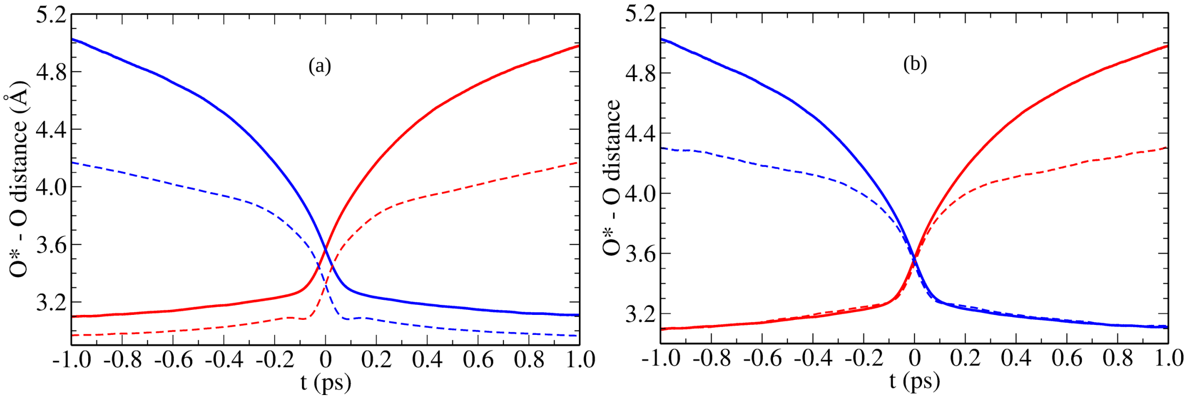

Figure S17: (a) Change in the O*–Oa (red) and O*–Ob (blue) distances, see the text and figure insets, during solvation water exchange (SWE) events for confined monolayer (solid) and bulk (dashed) water. (a) Change in the O*–Oa (red) and O*–Ob (blue) distances, see the text and figure insets, during solvation water exchange (SWE) events for confined monolayer (solid) and interfacial (dashed) water.

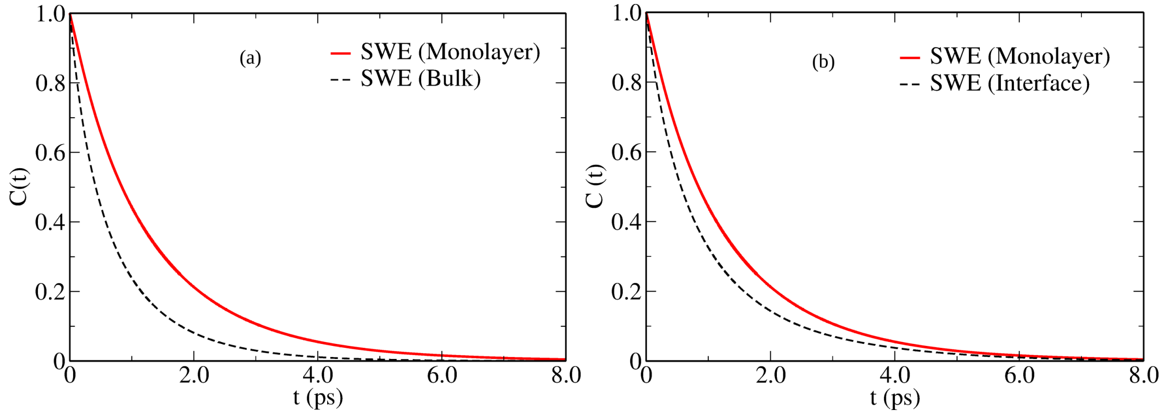

Figure S18: (a) Transition (jump) time correlation functions for solvation water exchange (SWE) events as described in the text for confined monolayer and bulk water. (b) Transition (jump) time correlation functions for solvation water exchange (SWE) events as described in the text for confined monolayer and interfacial water.

of the bulk. This increase is attributed to the presence of dangling bonds, which make the solvation shell more flexible. Using the CTRW model,[22,23] see text, we calculate the diffusion coefficient due to the SWE event as $D_{\rm SWE} = \delta^2_{\rm O*}/4\tau_{\rm SWE}$. From the square of the xy-component of the displacement amplitude (0.51 Å^2) and the transition time (1.01 ps), the SWE contribution to diffusion at the interface is found to be $D_{\rm SWE} = 1.26\times\ 10^{-5}$ cm^{2}s^{-1}. The diffusion due to solvation water exchange (or H-bond exchange) is found to be lower than that of the bulk, which is consistent with earlier observations.[29,30] Although the interface is more heterogeneous than the bulk due to the presence of dangling bond

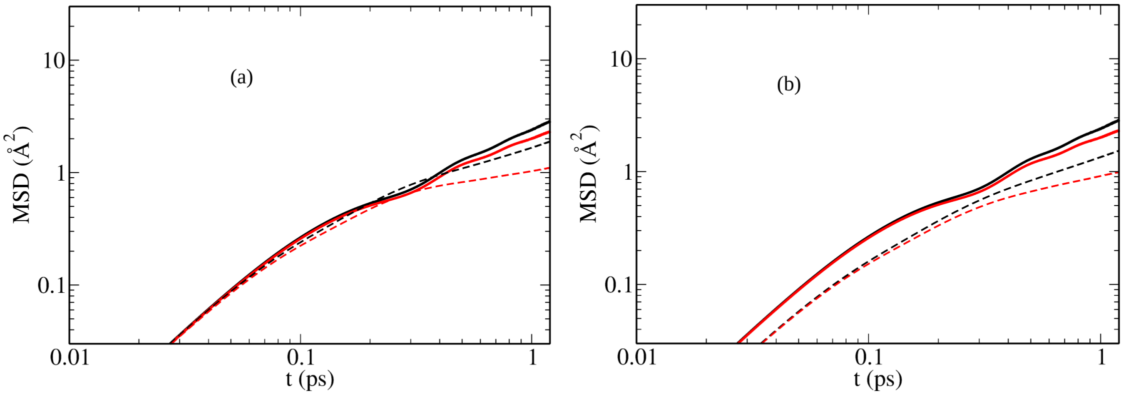

Figure S19: (a) Mean-square displacement (averaged over all molecules) in confined monolayer (black solid) and bulk (black dashed) water and MSD of water molecules in a solvation shell in confined monolayer (red solid) and bulk (black dashed) water. (b) Mean-square displacement (averaged over all molecules) in confined monolayer (black solid) and interfacial (black dashed) water and MSD of water molecules in a solvation shell in confined monolayer (red solid) and interfacial (black dashed) water.

(DB) states, solvation water (or H-bond) exchange remains the primary factor influencing overall diffusion, much like in the bulk. Hence, this dangling bond-dominated dynamics is entirely unique to water in monolayer confinement, contrasting sharply with the behavior observed in both bulk water and at interfaces.

References

(1) Marx, D.; Hutter, J. *Ab initio molecular dynamics: Basic theory and advanced methods*; Cambridge University Press: Cambridge, 2009.

(2) Ruiz-Barragan, S.; Sebastiani, F.; Schienbein, P.; Abraham, J.; Schwaab, G.; Nair, R. R.; Havenith, M.; Marx, D. Nanoconfinement effects on water in narrow graphene-based slit pores as revealed by THz spectroscopy. *Phys. Chem. Chem. Phys.* **2022**, *24*, 24734–24747.

(3) Ruiz-Barragan, S.; Muñoz Santiburcio, D.; Körning, S.; Marx, D. Quantifying anisotropic dielectric response properties of nanoconfined water within graphene slit pores. *Phys. Chem. Chem. Phys.* **2020**, *22*, 10833–10837.

(4) Das, B.; Ruiz-Barragan, S.; Marx, D. Deciphering the properties of nanoconfined aqueous solutions by vibrational sum frequency generation spectroscopy. *J. Phys. Chem. Lett.* **2023**, *14*, 1208–1213.

(5) Wang, Y.; Tang, F.; Yu, X.; Chiang, K.-Y.; Yu, C.-C.; Ohto, T.; Chen, Y.; Nagata, Y.; Bonn, M. Interfaces govern structure of angstrom-scale confined water. 2024; `https://arxiv.org/abs/2310.10354`, Submitted on 16 Oct 2023; Accessed: 2024-07-20.

(6) Gekle, S.; Netz, R. R. Anisotropy in the dielectric spectrum of hydration water and its relation to water dynamics. *J. Chem. Phys.* **2012**, *137*, 104704.

(7) Schlaich, A.; Knapp, E. W.; Netz, R. R. Water dielectric effects in planar confinement. *Phys. Rev. Lett.* **2016**, *117*, 048001.

(8) Hammer, B.; Hansen, L. B.; Nørskov, J. K. Improved adsorption energetics within density-functional theory using revised Perdew-Burke-Ernzerhof functionals. *Phys. Rev. B* **1999**, *59*, 7413.

(9) Grimme, S.; Antony, J.; Ehrlich, S.; Krieg, H. A consistent and accurate ab ini-

tio parametrization of density functional dispersion correction (DFT-D) for the 94 elements H-Pu. *J. Chem. Phys.* **2010**, *132*, 154104.

(10) Forster-Tonigold, K.; Groß, A. Dispersion corrected RPBE studies of liquid water. *J. Chem. Phys.* **2014**, *141*, 064501.

(11) Goedecker, S.; Teter, M.; Hutter, J. Separable dual-space Gaussian pseudopotentials. *Phys. Rev. B* **1996**, *54*, 1703.

(12) Hartwigsen, C.; Gœdecker, S.; Hutter, J. Relativistic separable dual-space Gaussian pseudopotentials from H to Rn. *Phys. Rev. B* **1998**, *58*, 3641.

(13) Krack, M. Pseudopotentials for H to Kr optimized for gradient-corrected exchange-correlation functionals. *Theor. Chem. Acc.* **2005**, *114*, 145–152.

(14) Abascal, J. L. F.; Vega, C. A general purpose model for the condensed phases of water: TIP4P/2005. *J. Chem. Phys.* **2005**, *123*, 234505.

(15) VandeVondele, J.; Krack, M.; Mohamed, F.; Parrinello, M.; Chassaing, T.; Hutter, J. Quickstep: Fast and accurate density functional calculations using a mixed Gaussian and plane waves approach. *Comput. Phys. Commun.* **2005**, *167*, 103–128.

(16) Hutter, J.; Iannuzzi, M.; Schiffmann, F.; VandeVondele, J. CP2K: atomistic simulations of condensed matter systems. *Wiley Interdiscip. Rev. Comput. Mol. Sci.* **2014**, *4*, 15–25.

(17) Kühne, T. D. et al. CP2K: An electronic structure and molecular dynamics software package - Quickstep: Efficient and accurate electronic structure calculations. *J. Chem. Phys.* **2020**, *152*, 194103.

(18) CP2K Open Source Molecular Dynamics. `https://www.cp2k.org/`, Accessed: 2021-11-01.

(19) Das, B.; Sharma, B.; Chandra, A. Effects of tert-butyl alcohol on water at the liquid–vapor interface: Structurally bulk-like but dynamically slow interfacial water. *J. Phys. Chem. C* **2018**, *122*, 9374–9388.

(20) Laage, D.; Hynes, J. T. On the molecular mechanism of water reorientation. *J. Phys. Chem. B* **2008**, *112*, 14230–14242.

(21) Stirnemann, G.; Rossky, P. J.; Hynes, J. T.; Laage, D. Water reorientation, hydrogen-bond dynamics and 2D-IR spectroscopy next to an extended hydrophobic surface. *Faraday Discuss.* **2010**, *146*, 263–281.

(22) Gomez, A.; Piskulich, Z. A.; Thompson, W. H.; Laage, D. Water diffusion proceeds via a hydrogen-bond jump exchange mechanism. *J. Phys. Chem. Lett.* **2022**, *13*, 4660–4666.

(23) Montroll, E. W.; Weiss, G. H. Random walks on lattices. II. *J. Math. Phys.* **1965**, *6*, 167–181.

(24) Laage, D.; Hynes, J. T. Do more strongly hydrogen-bonded water molecules reorient more slowly? *Chem. Phys. Lett.* **2006**, *433*, 80–85.

(25) Rezus, Y. L. A.; Bakker, H. J. Observation of immobilized water molecules around hydrophobic groups. *Phys. Rev. Lett.* **2007**, *99*, 148301.

(26) Bakulin, A. A.; Pshenichnikov, M. S.; Bakker, H. J.; Petersen, C. Hydrophobic molecules slow down the hydrogen-bond dynamics of water. *J. Phys. Chem. A* **2011**, *115*, 1821–1829.

(27) Laage, D.; Hynes, J. T. A molecular jump mechanism of water reorientation. *Science* **2006**, *311*, 832–835.

(28) Laage, D.; Thompson, W. H. Reorientation dynamics of nanoconfined water: Power-law decay, hydrogen-bond jumps, and test of a two-state model. *J. Chem. Phys.* **2012**, *136*.

(29) Liu, P.; Harder, E.; Berne, B. J. Hydrogen-bond dynamics in the air- water interface. *J. Phys. Chem. B* **2005**, *109*, 2949–2955.

(30) Ni, Y.; Gruenbaum, S. M.; Skinner, J. L. Slow hydrogen-bond switching dynamics at the water surface revealed by theoretical two-dimensional sum-frequency spectroscopy. *Proc. Natl. Acad. Sci. U.S.A.* **2013**, *110*, 1992–1998.
